# Sphingadienine-1-phosphate levels are regulated by a novel glycoside hydrolase family 1 glucocerebrosidase widely distributed in seed plants

**DOI:** 10.1016/j.jbc.2021.101236

**Published:** 2021-09-23

**Authors:** Jinichiro Koga, Makoto Yazawa, Koji Miyamoto, Emi Yumoto, Tomoyoshi Kubota, Tomoko Sakazawa, Syun Hashimoto, Masaki Sato, Hisakazu Yamane

**Affiliations:** 1 Department of Biosciences, School of Science and Engineering, Teikyo University, Tochigi, Japan; 2 Advanced Instrumental Analysis Center, Teikyo University, Tochigi, Japan

**Keywords:** glucocerebrosidase, ceramide, glucosylceramide, rice, long-chain base phosphate, sphingadienine-1-phosphate, sphingosine-1-phosphate, stomatal closure, drought tolerance, abscisic acid, ABA, abscisic acid, ABA-GE, glucose-conjugated abscisic acid, Cer, ceramide, C6-NBD-GluCer, N-[6-[(7-nitro-2-1,3-benzoxadiazol-4-yl)amino]hexanoyl]-d-glucosyl-β1-1′-sphingosine, d19:2(4*E*,8*E*,9Me)-C16h:0-GluCer, (4*E*,8*E*)-*N*-d-2′-hydroxypalmitoyl-1-*O*-β-d-glucopyranosyl-9-methyl-4,8-sphingadienine, d19:2(4*E*,8*E*,9Me)-C16h:1-GluCer, (4*E*,8*E*)-*N*-d-2′-hydroxy-(*E*)-3′-hexadecenoyl-1-*O*-β-d-glucopyranosyl-9-methyl-4,8-sphingadienine, d19:2(4*E*,8*E*,9Me)-C18h:1-GluCer, (4*E*,8*E*)-*N*-d-2′-hydroxy-(*E*)-3′-octadecenoyl-1-*O*-β-d-glucopyranosyl-9-methyl-4,8-sphingadienine, GalCer, galactosylceramide, GCase, glucocerebrosidase, GH, glycoside hydrolase family, GIPC, glycosylinositol phosphoceramide, Glu, glucose, GluCer, glucosylceramide, LacCer, lactosylceramide, LCB, long-chain base, LCBP, long-chain base phosphate, LC-ESI-MS/MS, liquid chromatography electrospray ionization–tandem mass spectrometry, MALDI-TOF MS, matrix-assisted laser desorption ionization–time of flight mass spectrometry, MRM, multiple reaction monitoring, PMF, peptide mass fingerprinting, pNP-β-glucoside, *p*-nitrophenyl-β-glucopyranoside, SDS-PAGE, sodium dodecyl sulfate–polyacrylamide gel electrophoresis, sphingadienine, (4*E*,8*Z*)-sphingadienine, sphingadienine-1-phosphate, (4*E*,8*Z*)-sphingadienine-1-phosphate

## Abstract

Long-chain base phosphates (LCBPs) such as sphingosine-1-phosphate and phytosphingosine-1-phosphate function as abscisic acid (ABA)-mediated signaling molecules that regulate stomatal closure in plants. Recently, a glycoside hydrolase family 1 (GH1) β-glucosidase, Os3BGlu6, was found to improve drought tolerance by stomatal closure in rice, but the biochemical functions of Os3BGlu6 have remained unclear. Here we identified Os3BGlu6 as a novel GH1 glucocerebrosidase (GCase) that catalyzes the hydrolysis of glucosylceramide to ceramide. Phylogenetic and enzymatic analyses showed that GH1 GCases are widely distributed in seed plants and that pollen or anthers of all seed plants tested had high GCase activity, but activity was very low in ferns and mosses. Os3BGlu6 had high activity for glucosylceramides containing (4*E*,8*Z*)-sphingadienine, and GCase activity in leaves, stems, roots, pistils, and anthers of Os3BGlu6-deficient rice mutants was completely absent relative to that of wild-type rice. The levels of ceramides containing sphingadienine were correlated with GCase activity in each rice organ and were significantly lower in Os3BGlu6-deficient rice mutants than in the wild type. The levels of LCBPs synthesized from ceramides, especially the levels of sphingadienine-1-phosphate, were also correlated with GCase activity in each rice organ and were significantly lower in Os3BGlu6-deficient rice mutants than in the wild type. These results indicate that Os3BGlu6 regulates the level of ceramides containing sphingadienine, influencing the regulation of sphingadienine-1-phosphate levels and subsequent improvement of drought tolerance *via* stomatal closure in rice.

Glucocerebrosidase (GCase; EC 3.2.1.45) catalyzes the hydrolysis of glucosylceramide to glucose and ceramide (Fig. 1*A*) and is critical in regulating animal physiology. Four human GCases are known: lysosomal GCase (GBA1, glycoside hydrolase family [GH] 30), cytosolic GCase (GBA2, GH116), cytosolic GCase (GBA3, GH1), and intestinal lactase/phlorizin hydrolase (GH1). Gaucher's disease, the most prevalent lysosomal storage disorder, is caused by mutations in the gene encoding a lysosomal enzyme GBA1 (GH30), leading to glucosylceramide accumulation in the liver, spleen, and bone marrow and to severe complications such as thrombocytopenia and skeletal deterioration (1, 2). Therefore, a recombinant human GBA1 has been developed as an enzyme replacement therapy (3, 4). Animal GCases have been extensively studied, but in plants, no GCases were found until 2020, and their physiological functions remain unclear. In 2020, the first plant GCase, *At*GCD3, was identified from *Arabidopsis thaliana* as a homolog of human cytosolic GBA2 (GH116) (5). However, *At*GCD3 activity has not been detected in *A. thaliana*, and ceramide levels in *At*GCD3*-*deficient mutants were not significantly different compared with the wild type. Thus, a GH116 GCase such as *At*GCD3 seems unlikely to function in plants.

**Figure 1:**
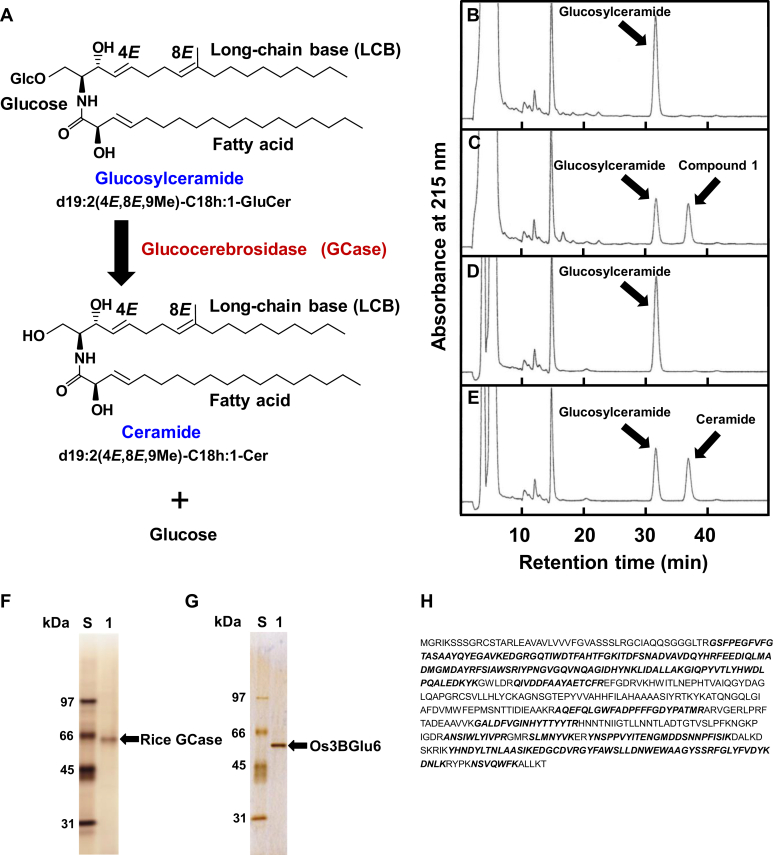
**Purification and identification of rice GCase.***A*, conversion of glucosylceramide to ceramide by GCase. HPLC chromatograms (*B*) before and (*C*) after incubation with a fungal glucosylceramide [d19:2(4*E*,8*E*,9Me)-C18h:1-GluCer] and the crude enzyme extracted from rice leaves. HPLC chromatograms (*D*) before and (*E*) after incubation with the fungal glucosylceramide and recombinant Os3BGlu6 protein extracts expressed in *E. coli*. *F*, SDS-PAGE of purified GCase from rice leaves. Lane S, standard proteins; lane 1, purified GCase from rice leaves. *G*, SDS-PAGE of purified Os3BGlu6 from *E. coli* expressing *Os3BGlu6* gene. Lane S, standard proteins; lane 1, purified Os3BGlu6 from *E. coli* expressing *Os3BGlu6* gene. *H*, Amino acid sequence of Os3BGlu6. *Bold italic* characters indicate peptides with molecular mass identical to that of peptide fragments from the 62-kDa protein. SDS-PAGE, sodium dodecyl sulfate–polyacrylamide gel electrophoresis.

Os3BGlu6 was previously isolated from rice as a GH1 β-glucosidase, and the three-dimensional structure was determined by X-ray crystallography, but its true substrate has not been revealed yet (6). Recently, Wang *et al.* (7) found that a disruption of *Os3BGlu6* gene in rice plants led to decrease in abscisic acid (ABA) levels and to stomatal opening in the leaves, making them sensitive to drought, whereas a reversion of *Os3BGlu6* gene to the Os3BGlu6-deficient mutant led to stomatal closure in the leaves, resulting in resistance to drought. Thus, they proposed that Os3BGlu6 elevates cellular ABA levels, thereby improving drought tolerance by stomatal closure in rice plants, but the biochemical functions of Os3BGlu6 have remained unclear.

In the present study, we identified Os3BGlu6 as a novel GH1 GCase that regulates the level of ceramides. Previous studies have revealed that long-chain base phosphates (LCBPs) such as sphingosine-1-phosphate and phytosphingosine-1-phosphate function as ABA-mediated signaling molecules that regulate stomatal closure in plants (8, 9, 10, 11). Thus, we speculated that LCBP levels increase after Os3BGlu6 activation and the LCBPs might act as signaling molecules for stomatal closure in rice, because LCBPs are synthesized from ceramides hydrolyzed by GCase. To explore this possibility, we examined the biochemical properties of Os3BGlu6 and the regulation of LCBP levels by Os3BGlu6 using Os3BGlu6-deficient rice mutants.

## Results

### Identification and purification of rice GCase

When plants interact with pathogens, they protect themselves through various defenses such as antimicrobial compounds called phytoalexins, pathogenesis-related proteins, and hypersensitive cell death (12, 13). These defense responses are induced by molecules from the pathogens called elicitors (14). We isolated five novel phytoalexins from rice leaves infected with the pathogenic fungus *Pyricularia oryzae*, designated phytocassanes A, B, C, D, and E (15, 16). The most active elicitors of phytoalexin production in rice were isolated from *P*. *oryzae*, and their structures were identified as cerebrosides A and C (Fig. 1*A*), fungal glucosylceramides (17, 18). Treatment of rice leaves with the fungal glucosylceramide induced the production of phytoalexins, pathogenesis-related proteins, hypersensitive cell death, and complete resistance to subsequent infection by virulent pathogens (18, 19, 20). Previous studies suggest that the hypersensitive cell death in plants is a type of programmed cell death and that ceramides induce programmed cell death in *A. thaliana* (21, 22, 23). Thus, we hypothesized that fungal glucosylceramide might be hydrolyzed to ceramide by rice GCase and that this liberated ceramide might induce defense responses.

When a fungal glucosylceramide [d19:2(4*E*,8*E*,9Me)-C18h:1-GluCer] (Fig. 1*A*) was incubated with an enzyme solution extracted from rice leaves, a new peak designated compound 1 appeared in a HPLC chromatogram (Fig. 1, *B* and *C*). Therefore, compound 1 was purified and subjected to a liquid chromatography electrospray ionization–tandem mass spectrometry (LC-ESI-MS/MS). The precursor ion, *m*/*z* 592.6 [M+H]^+^ and the product ion, m/z 276.2 of compound 1 in LC-ESI-MS/MS were identical to those of a standard ceramide [d19:2(4*E*,8*E*,9Me)-C18h:1-Cer]. This result indicates that the enzyme solution extracted from rice leaves has GCase activity. Therefore, two-step chromatographic fractionation with HiTrap Q HP anion exchange and HiTrap SP HP cation exchange chromatography was used to purify the enzyme to apparent homogeneity as a single band by sodium dodecyl sulfate–polyacrylamide gel electrophoresis (SDS-PAGE) with mobility corresponding to an apparent molecular mass of 62-kDa (Fig. 1*F*).

### Molecular cloning and characterization of rice GCase gene

The 62-kDa band of the purified rice GCase on SDS-PAGE was excised from the gel, treated with trypsin, and the products of tryptic digestion were analyzed by matrix-assisted laser desorption ionization–time of flight mass spectrometry (MALDI-TOF MS). This analysis showed that the molecular mass of nine peptide fragments obtained from the 62-kDa protein was identical to that of the peptide sequence of Os3BGlu6 that was previously isolated and found to be an uncharacterized GH1 β-glucosidase isolated from rice (6) (Figs. 1*H* and S1, Tables S2 and S3). To confirm whether the purified rice GCase is Os3BGlu6, the Os3BGlu6 cDNA was cloned, and the recombinant protein was expressed in *E. coli*. Glucosylceramide was efficiently converted to ceramide by the recombinant protein (Fig. 1, *D* and *E*), suggesting that Os3BGlu6 functions as a novel GCase belonging to GH1, different from *At*GCD3 previously identified as GH116 GCase from *A. thaliana*.

By two-step chromatographic fractionation using HiTrap Q HP anion exchange and HiTrap SP HP cation exchange chromatography, we purified the crude extract containing the recombinant Os3BGlu6 in *E. coli* to apparent homogeneity as a single band by SDS-PAGE with mobility corresponding to an apparent molecular mass of 56 kDa (Fig. 1*G*). The *K*_m_ and *K*_cat_ values for the fungal glucosylceramide [d19:2(4*E*,8*E*,9Me)-C16h:0-GluCer] of the enzyme were 215.0 ± 10.2 μM and 134.4 ± 8.8 s^−1^, respectively. The *K*_cat_ value of the recombinant Os3BGlu6 (134.4 ± 8.8 s^−1^) was very similar to that of Os3BGlu6 purified from rice plants (144.6 ± 7.6 s^−1^), whereas the *K*_m_ value of the recombinant Os3BGlu6 (215.0 ± 10.2 μM) was higher than that of Os3BGlu6 from rice plants (38.1 ± 2.4 μM) (Table 1). Since the recombinant Os3BGlu6 expressed in *E. coli* is not glycosylated, the sugar chains bound to Os3BGlu6 might be involved in the affinity for the substrate.

**Table 1:** Kinetics of Os3BGlu6 for hydrolysis of various substrates

Enzyme	*K*_m_ (μM)	*K*_cat_ (s^−1^)	*K*_cat_/*K*_m_ (s^−1^/μM)
Substrate			
Os3BGlu6			
Animal glucosylceramides			
d18:1(4*E*)-C8:0-GluCer	47.5 ± 3.8	156.1 ± 8.6	3.29 ± 0.15
d18:1(4*E*)-C12:0-GluCer	46.1 ± 5.1	121.1 ± 4.4	2.64 ± 0.19
Plant glucosylceramides			
d18:2(4*E*,8*Z*)-C16h:0-GluCer	19.8 ± 0.8	115.8 ± 2.9	5.85 ± 0.19
d18:2(4*E*,8*E*)-C16h:0-GluCer	50.0 ± 2.4	145.8 ± 4.7	2.92 ± 0.05
Fungal glucosylceramides			
d19:2(4*E*,8*E*,9Me)-C16h:0-GluCer	38.1 ± 2.4	144.6 ± 7.6	3.79 ± 0.19
d19:2(4*E*,8*E*,9Me)-C16h:1-GluCer	41.1 ± 4.3	151.0 ± 9.1	3.70 ± 0.28
Synthetic β-glucosides			
*p*NP-β-glucoside	3825.3 ± 201.2	285.1 ± 10.3	0.075 ± 0.005
C6-NBD-GluCer	57.1 ± 7.9	112.1 ± 11.8	1.97 ± 0.12
Klotho-related protein (human GBA3)			
Animal glucosylceramide			
d18:1(4*E*)-C18:0-GluCer	13.7 ± 1.4	0.0072 ± 0.0002	0.0005 ± 0.0000
Synthetic glucosylceramide			
C6-NBD-GluCer	4.64 ± 0.24	0.121 ± 0.006	0.0262 ± 0.0005

Os3BGlu6 purified from rice plants and various concentrations of substrates were incubated in 0.2 ml of Buffer J at 37 °C for 15 min. The kinetic parameters were determined from Hanes–Woolf plots. The results are means ± SD of five experiments. Values for Klotho-related protein (human GBA3) are from Hayashi *et al.* (30).

Abbreviations: C6-NBD-GluCer, N-[6-[(7-nitro-2-1,3-benzoxadiazol-4-yl)amino]hexanoyl]-d-glucosyl-β1-1′-sphingosine; *p*NP-β-glucoside, *p*-nitrophenyl-β-glucopyranoside.

### Biochemical characterization of Os3BGlu6

We examined biochemical characteristics of Os3BGlu6 purified from rice plants. To confirm whether Os3BGlu6 specifically recognizes glucosylceramide structure, we tested a number of sphingolipid derivatives as substrates for Os3BGlu6 (Table 2). Among the compounds tested, galactosylceramide and lactosylceramide were hydrolyzed at a rate of 5.8% and 3.4% of the hydrolysis of glucosylceramide, respectively. However, ganglioside GM_3_ was not hydrolyzed. The *K*_cat_/*K*_m_ value for the fungal glucosylceramide [d19:2(4*E*,8*E*,9Me)-C16h:0-GluCer] of the enzyme was 3.79 ± 0.19 s^−1^/μM, about 50 times higher than for *p*-nitrophenyl-β-glucopyranoside (*p*NP-β-glucoside) (0.075 ± 0.005 s^−1^/μM) (Table 1). Furthermore, Seshadri *et al.* (6) showed that the *K*_cat_/*K*_m_ value for *p*NP-β-glucoside of Os3BGlu6 was higher than that for natural β-glucoside compounds such as *n*-octyl-β-d-glucoside, *n*-heptyl-β-d-glucoside, laminaribiose, laminaritriose, cellobiose, sophorose, and gentiobiose. These results presented above clearly show that Os3BGlu6 specifically recognizes the glucosylceramide structure.

**Table 2:** Substrate specificity of Os3BGlu6

Substrate	Sugar	Fatty acid	Long-chain base	Relative activity^a^ (%)
Animal sphingolipids				
Sphingolipids containing sphingenine				
d18:1(4*E*)-C18:0-GM_3_	NeuAc-Gal-Glu	18:0	d18:1(4*E*)	ND
d18:1(4*E*)-C8:0-LacCer	Lac	8:0	d18:1(4*E*)	3.4 ± 0.3
d18:1(4*E*)-C8:0-GalCer	Gal	8:0	d18:1(4*E*)	5.8 ± 0.5
d18:1(4*E*)-C8:0-GluCer	Glu	8:0	d18:1(4*E*)	100.0 ± 4.3
d18:1(4*E*)-C12:0-GluCer	Glu	12:0	d18:1(4*E*)	82.9 ± 3.8
d18:1(4*E*)-C18:0-GluCer	Glu	18:0	d18:1(4*E*)	50.4 ± 5.2
Plant glucosylceramides				
Glucosylceramides containing sphingadienine				
d18:2(4*E*,8*Z*)-C16h:0-GluCer	Glu	16h:0	d18:2(4*E*,8*Z*)	147.7 ± 3.8
d18:2(4*E*,8*E*)-C16h:0-GluCer	Glu	16h:0	d18:2(4*E*,8*E*)	96.1 ± 2.6
d18:2(4*E*,8*Z*)-C18h:0-GluCer	Glu	18h:0	d18:2(4*E*,8*Z*)	143.3 ± 6.8
d18:2(4*E*,8*Z*)-C20h:0-GluCer	Glu	20h:0	d18:2(4*E*,8*Z*)	127.8 ± 13.0
d18:2(4*E*,8*Z*)-C22h:0-GluCer	Glu	22h:0	d18:2(4*E*,8*Z*)	106.8 ± 7.3
d18:2(4*E*,8*Z*)-C24h:0-GluCer	Glu	24h:0	d18:2(4*E*,8*Z*)	97.2 ± 11.3
Glucosylceramides containing 4-hydroxysphingenine				
t18:1(8*Z*)-C22h:0-GluCer	Glu	22h:0	t18:1(8*Z*)	43.1 ± 3.5
t18:1(8*Z*)-C24h:0-GluCer	Glu	24h:0	t18:1(8*Z*)	35.3 ± 4.2
Fungal glucosylceramides				
Glucosylceramides containing sphingadienine				
d19:2(4*E*,8*E*,9Me)-C16h:0-GluCer	Glu	16h:0	d19:2(4*E*,8*E*,9Me)	116.7 ± 5.1
d19:2(4*E*,8*E*,9Me)-C16h:1-GluCer	Glu	16h:1	d19:2(4*E*,8*E*,9Me)	117.7 ± 6.9
d19:2(4*E*,8*E*,9Me)-C18h:1-GluCer	Glu	18h:1	d19:2(4*E*,8*E*,9Me)	113.2 ± 5.9

Abbreviations: 16:0, hexadecanoic acid; d18:1(4*E*), (4*E*)-4-sphingenine; d18:2(4*E*,8*E*), (4*E*,8*E*)-4,8-sphingadienine; 16h:1, 2-hydroxy-(*E*)-3-hexadecenoic acid; 18h:0: 2-hydroxyoctadecanoic acid; 18h:1: 2-hydroxy-(*E*)-3-octadecenoic acid; 20h:0, 2-hydroxyicosanoic acid; 22h:0, 2-hydroxydocosanoic acid; 24h:0, 2-hydroxytetraicosanoic acid; Cer, ceramide; d19:2(4*E*,8*E*,9Me), (4*E*,8*E*)-9-methyl-4,8-sphingadienine; Gal, galactose; GalCer, galactosylceramide; Glu, glucose; GluCer, glucosylceramide; Lac, lactose; NeuAc, acetylneuraminic acid; LacCer, lactosylceramide; ND, not detectable; t18:1(8*Z*), (8*Z*)-4-hydroxy-8-sphingenine.

a Os3BGlu6 purified from rice plants and 12.5 μM of substrate were incubated in 0.2 ml of Buffer J at 37 °C for 15 min. The results are means ± SD of five experiments.

As shown in Table 2, GCase activity was lower for glucosylceramides with the long-chain base (LCB)[d18:1(4*E*)], with increasing fatty acid chain length when 8:0, 12:0, and 18:0 were compared, so glucosylceramides with the fatty acid of the same chain length were used as substrates to compare GCase activity for each glucosylceramide described below. The *K*_cat_/*K*_m_ value for fungal glucosylceramides of Os3BGlu6 was not much different from that for plant glucosylceramides (Table 1). It seems likely that Os3BGlu6 does not specifically recognize a methyl group of the LCB, a unique structure of fungal glucosylceramides (Fig. 1*A*). On the other hand, GCase activity for plant glucosylceramides containing sphingadienine [d18:2(4*E*,8*Z*)-C22∼24h:0-GluCer], main glucosylceramides in rice (24, 25), was two or three times higher than for plant glucosylceramides containing 4-hydroxysphingenine [t18:1(8*Z*)-C22∼24h:0-GluCer] (Table 2). Also, GCase activity for plant glucosylceramide containing sphingadienine [d18:2(4*E*,8*Z*)-C18h:0-GluCer] was about three times higher than for animal glucosylceramide containing sphingenine [d18:1(4*E*)-C18:0-GluCer] (Table 2). This result indicates that Os3BGlu6 has high activity for glucosylceramides containing sphingadienine. Furthermore, the *K*_cat_/*K*_m_ value for d18:2(4*E*,8*Z*)-C16h:0-GluCer of Os3BGlu6 was 5.85 ± 0.19 s^−1^/μM, about two times higher than for d18:2(4*E*,8*E*)-C16h:0-GluCer (2.92 ± 0.05 s^−1^/μM), indicating that Os3BGlu6 specifically recognizes the 8*Z* double bond in the LCB (Table 1). These results presented above show that Os3BGlu6 has highest activity for glucosylceramides containing (4*E*,8*Z*)-sphingadienine (sphingadienine).

### Regulation of ceramide levels by Os3BGlu6 in rice

**Figure 2:**
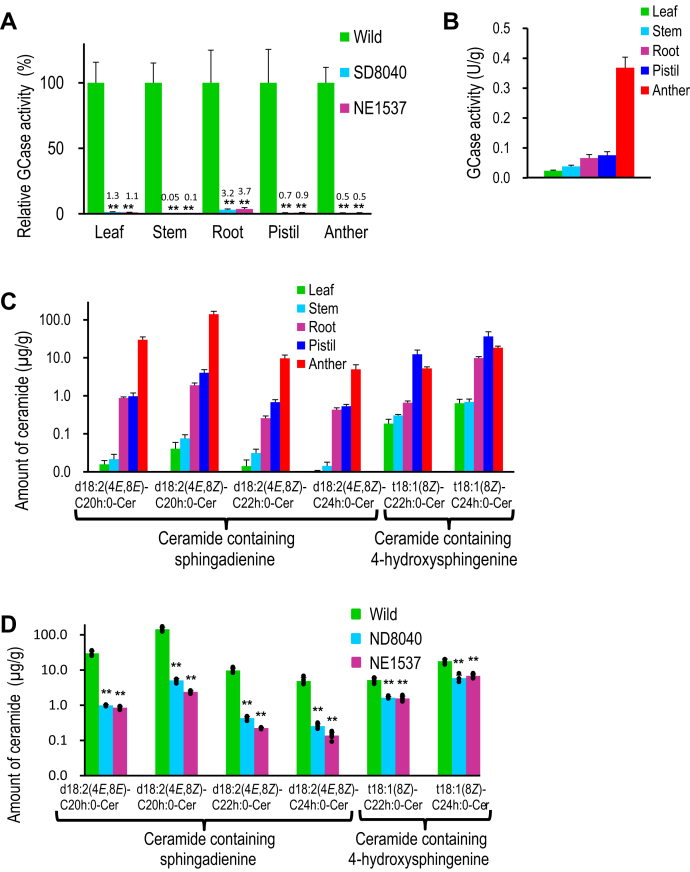
**GCase activity and amount of ceramides in rice**. *A*, GCase activity in organs of two Os3BGlu6-deficient rice mutants NE1537 and ND8040 relative to that in the wild type (set as 100%). *Asterisks* indicate a significant difference in GCase activity for either mutant compared to that for the wild type in Student's *t* test (∗∗*p* < 0.01, *n* = 3). *B*, GCase activity in rice organs. GCase activity is expressed as units (U) per fresh weight (g) of plant material (*n* = 3). *C*, amount of ceramides (μg/g fresh weight of plant material) in organs of rice (*n* = 3). *D*, amount of ceramides in anthers of Os3BGlu6-deficient rice mutants and the wild type. *Asterisks* indicate a significant difference in the amount of ceramide in anthers of either of Os3BGlu6-deficient rice mutants NE1537 and ND8040 compared to that of the wild type in Student's *t* test (∗∗*p* < 0.01, n = 5).

A homolog of *At*GCD3, the GH116 GCase from *A. thaliana*, was found in *Oryza sativa* (5), suggesting the possibility that the GCase activity in rice might be due to GH116 GCase as well as Os3BGlu6 (GH1 GCase). Therefore, to confirm whether GCase activity in rice is due to Os3BGlu6, we obtained two rice mutants deficient in Os3BGlu6, NE1537 and ND8040 (https://tos.nias.affrc.go.jp/index.html.ja), which have the retrotransposon *Tos17* inserted in the *Os3BGlu6* gene of rice. GCase activity in leaves, stems, roots, pistils, and anthers of the two rice Os3BGlu6-deficient mutants was completely absent relative to that of the wild type (Fig. 2*A*), suggesting that almost all GCase activity in rice is due to Os3BGlu6.

GCase activity in anthers was found to be 5–16 times higher than in other organs (Fig. 2*B*). Therefore, we examined the regulation of ceramide levels by Os3BGlu6 in rice. First, to confirm whether high amounts of ceramides are, indeed, liberated by Os3BGlu6, which has high activity in anthers, we measured the amount of ceramides in various rice organs using LC-ESI-MS/MS as described (26). As shown in Figure 2*C*, the total amounts of ceramides in anthers reached 0.208 mg/g, 4–231 times higher than in the other organs. In particular, ceramides containing sphingadienine such as d18:2(4*E*,8*E* or *Z*)-C20∼24h:0-Cer were most abundant in the anther compared with the other organs, but ceramides containing 4-hydroxysphingenine such as t18:1(8*Z*)-C22∼24h:0-Cer were most abundant in the pistil compared with the other organs (Fig. 2*C*). The amount of ceramides containing sphingadienine in each rice organ was correlated with the GCase activity in each rice organ (Fig. 2, *B* and *C*), suggesting that the level of ceramides containing sphingadienine might be regulated by Os3BGlu6.

To confirm whether the level of ceramides containing sphingadienine is regulated by Os3BGlu6, we measured the amount of ceramides in anthers of Os3BGlu6-deficient rice mutants by LC-ESI-MS/MS as described (26). The amounts of ceramides containing sphingadienine in anthers of Os3BGlu6-deficient rice mutants (NE1537 and ND8040) were 20–60 times lower than in the wild type, but the amounts of ceramides containing 4-hydroxysphingenine in the anthers were only 2–3 times lower than in the wild type (Fig. 2*D*). This result clearly shows that the level of ceramides containing sphingadienine is regulated by Os3BGlu6 in rice plants.

### Physiological role of Os3BGlu6 in rice

**Figure 3:**
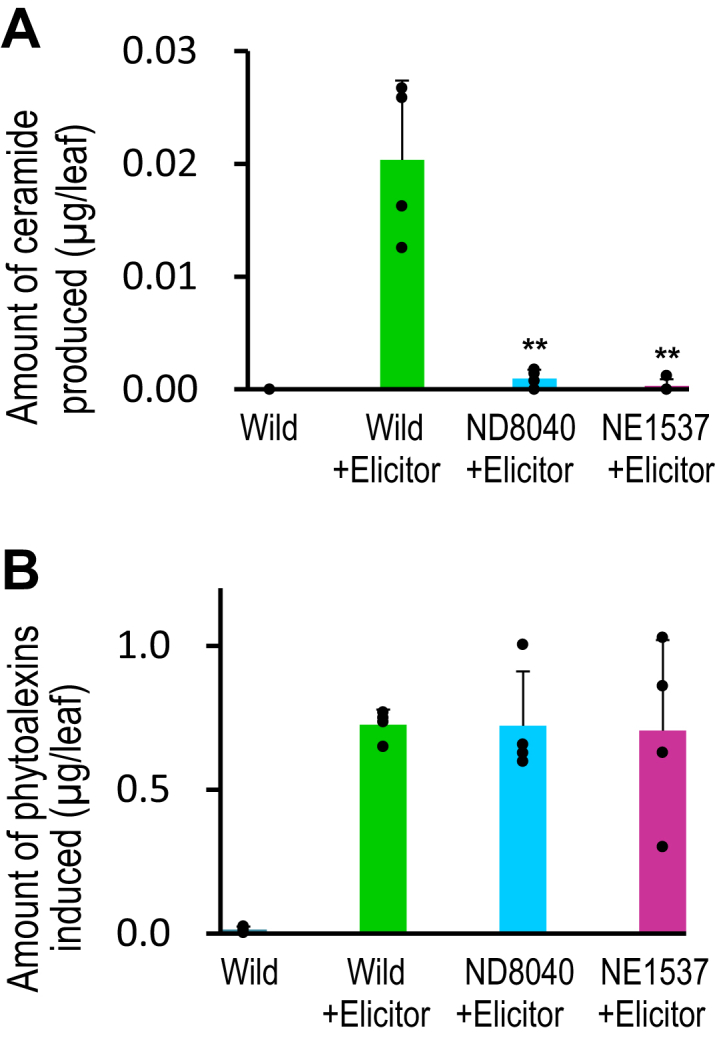
**Amount of ceramide and phytoalexins produced in Os3BGlu6-deficient rice mutants and the wild typeby treatment with fungal glucosylceramide elicitor**. Rice leaves were treated with 0.1% Tween 20 (control) or 0.1% Tween 20 plus 100 μM fungal glucosylceramide (elicitor), then harvested after 72 h to measure the amount of ceramide produced (*A*) and the total amount of phytoalexins (phytocassanes A–E plus momilactones A and B) (15, 16, 46) induced (*B*) using LC-ESI-MS/MS as described (26, 47). *Asterisks* indicate a significant difference in the amount of ceramide in leaves of elicitor-treated Os3BGlu6-deficient rice mutants NE1537 and ND8040 compared with that of the elicitor-treated wild type (Student's *t* test, ∗∗*p* < 0.01, *n* = 4).

Next, we examined the physiological role of Os3BGlu6 in rice. First, to verify the involvement of Os3BGlu6 in defense responses against pathogens, we examined whether the fungal glucosylceramide elicitor (18) is hydrolyzed to ceramide by Os3BGlu6 and whether the liberated ceramide induces the synthesis of phytoalexins (15, 16), one of the defense responses. As speculated, a ceramide derived from the fungal glucosylceramide appeared in the leaves of the wild type, but was barely detected in the leaves of two Os3BGlu6-deficient rice mutants (NE1537 and ND8040) (Fig. 3*A*). However, the amounts of phytoalexins induced in the leaves of Os3BGlu6-deficient rice mutants with the elicitor treatment were almost the same as those of the wild type with the elicitor treatment (Fig. 3*B*). These results indicate that the ceramide generated from the fungal glucosylceramide by Os3BGlu6 is not involved in the defense response such as phytoalexin induction. From these results and the previous enzymatic results that Os3BGlu6 does not specifically recognize a methyl group of the LCB, a unique structure on fungal glucosylceramide (Table 1), we speculated that Os3BGlu6 has a function other than the defense response against pathogens.

**Figure 4:**
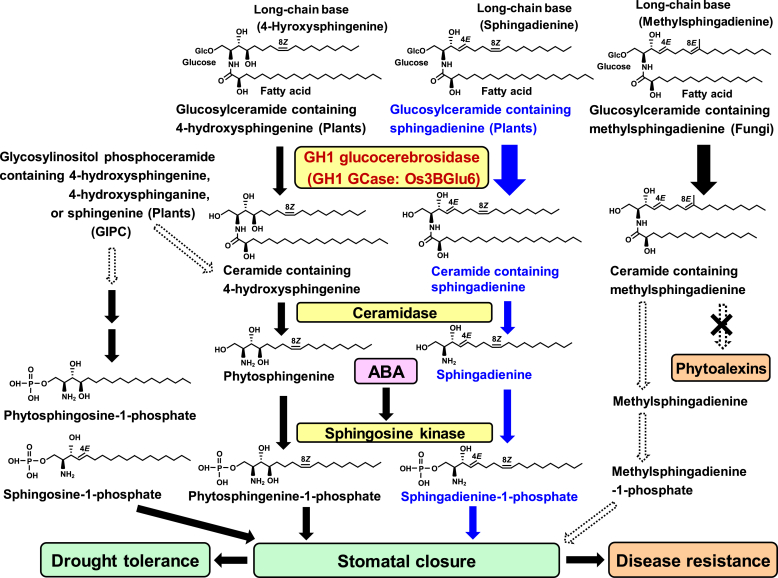
**Metabolic pathways of glucosylceramides in rice**. *Blue arrows* represent a pathway of stomatal closure induction by sphingagienine-1-phospate generated from glucosylceramides containing sphingadienine by Os3BGlu6. The *arrow* thickness for GH1 GCase shows the strength of GCase activity for each substrate. The *dotted arrows* represent hypothetical pathways.

Surprisingly, in 2020, Wang *et al.* (7) revealed that Os3BGlu6 in rice chloroplasts elevates cellular ABA levels, thereby improving drought tolerance *via* stomatal closure. Previous studies have revealed that LCBPs such as sphingosine-1-phosphate and phytosphingosine-1-phosphate function as ABA-mediated signaling molecules that regulate stomatal closure in plants (8, 9, 10, 11). Considering these facts, we speculated that LCBP levels increase after Os3BGlu6 activation and the LCBPs might act as signaling molecules for stomatal closure in rice, because LCBPs are synthesized from ceramides, and the level of ceramides is regulated by Os3BGlu6 as shown in Figure 4.

**Figure 5:**
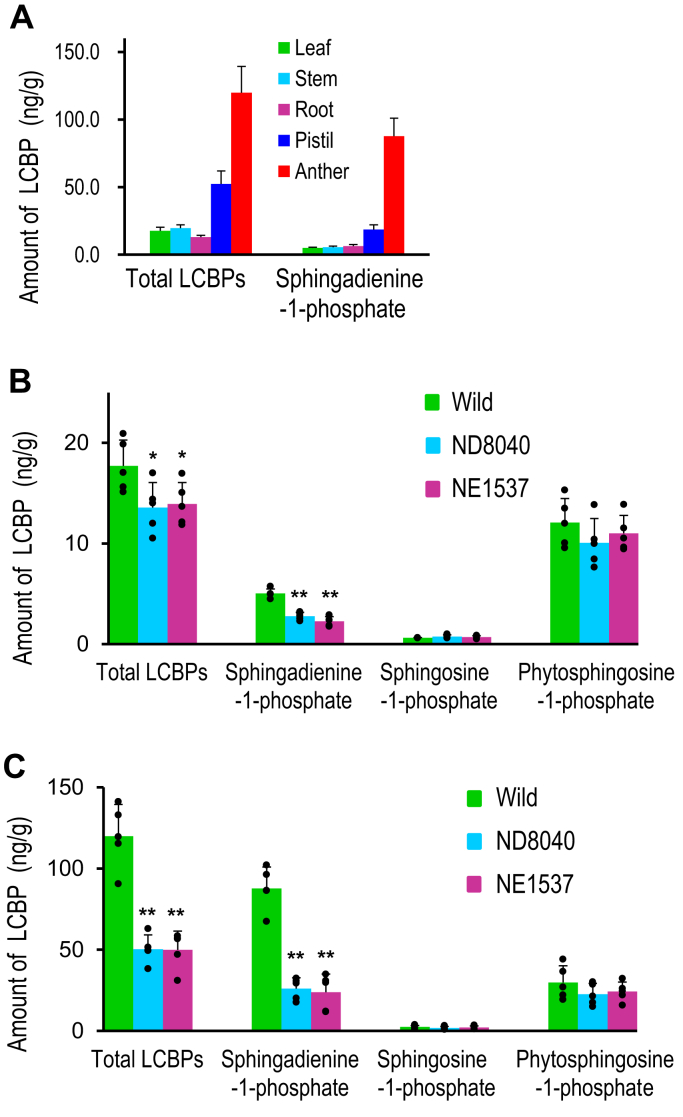
**Amount ofvariousLCBPsin rice**. *A*, amount of LCBPs in rice organs (ng/g fresh weight of plant material). *B*, amount of LCBPs in leaves and (*C*) anthers of Os3BGlu6-deficient rice mutants NE1537 and ND8040 and the wild type. *Asterisks* indicate a significant difference in the amount of LCBP in leaves or anthers of either mutant compared to that of the wild type in Student's *t* test (∗*p* < 0.05, ∗∗*p* < 0.01, *n* = 5). LCBP, long-chain base phosphate; sphingadienine-1-phosphate, (4*E*,8*Z*)-sphingadienine-1-phosphate.

To confirm whether the level of LCBPs in various rice organs is correlated with Os3BGlu6 activity, we measured the amount of LCBPs, which are available as reference standards, in each organ using LC-ESI-MS/MS as described (27, 28, 29). The total amounts of LCBPs in anthers reached 120 ng/g, 2 to 7 times higher than in the other organs (Fig. 5*A*). The levels of LCBPs, especially (4*E*,8*Z*)-sphingadienine-1-phosphate (sphingadienine-1-phosphate), were correlated with GCase activity in each organ (Figs. 2*B* and 5*A*), suggesting that the LCBP level might be regulated by Os3BGlu6. Next, to demonstrate whether the LCBP level is regulated by Os3BGlu6, we quantified the LCBP in the leaves and the anthers of Os3BGlu6-deficient rice mutants (NE1537 and ND8040) and found that the total amounts of LCBPs in each organ of the mutants were significantly lower than in the wild type (Fig. 5, *B* and *C*). Among LCBPs, the amounts of sphingadienine-1-phosphate in both mutants were significantly lower than in the wild type, but neither the amounts of phytosphingosine-1-phosphate nor sphingosine-1-phosphate differed between the mutants and the wild type (Fig. 5, *B* and *C*). Our findings and the study of Wang *et al.* (7) together indicate that Os3BGlu6 regulates the level of ceramide containing sphingadienine, influencing the regulation of sphingadienine-1-phosphate levels and subsequent improvement of drought tolerance via stomatal closure in rice (Fig. 4).

### Regulation of sphingadienine-1-phosphate levels by Os3BGlu6 in rice

**Figure 6:**
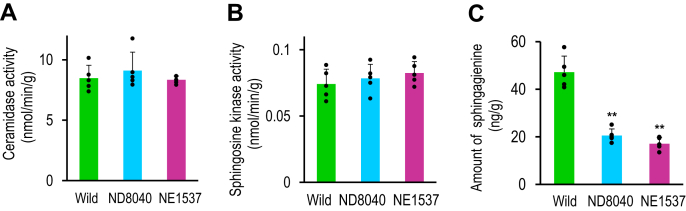
**Ceramidase, sphingosine kinase activity, and amount ofsphingadienine inleaves of Os3BGlu6-deficient rice mutants and the wild type.***A*, ceramidase and (*B*) sphingosine kinase activity in leaves of Os3BGlu6-deficient rice mutants NE1537 and ND8040 and the wild type. Enzyme activity is expressed as nmol/min/g fresh weight of plant material. *Asterisks* indicate a significant difference in the activity for either mutant compared to that for the wild type in Student's *t* test (∗*p* < 0.05, ∗∗*p* < 0.01, *n* = 5). *C*, amount of sphingagienine in leaves (ng/g fresh weight of plant material) of rice mutants NE1537 and ND8040 and the wild type. *Asterisks* indicate a significant difference in the amount of sphingadienine in leaves of either mutant compared to that of the wild type in Student's *t*-test (∗*p* < 0.05, ∗∗*p* < 0.01, *n* = 5). sphingadienine, (4*E*,8*Z*)-sphingadienine.

Ceramides containing sphingadienine are metabolized to sphingadienines by ceramidase, then sphingadienines are phosphorylated to sphingadienine-1-phosphate by sphingosine kinase (Fig. 4). Therefore, to examine whether ceramidase and sphingosine kinase, downstream enzymes of GCase, are regulated by Os3BGlu6, we measured the activity of these enzymes in Os3BGlu6-deficient rice mutants. The activity of ceramidase and sphingosine kinase did not differ significantly between the Os3BGlu6-deficient rice mutants (NE1537 and ND8040) and the wild type (Fig. 6, *A* and *B*), suggesting that these enzymes are not regulated by Os3BGlu6.

Next, we measured the amount of sphingadienine in Os3BGlu6-deficient rice mutants by LC-ESI-MS/MS. The amounts of sphingadienine in the leaves of Os3BGlu6-deficient rice mutants (NE1537 and ND8040) were significantly lower than those in the wild type (Fig. 6*C*). It has been reported that the *K*_m_ value for sphingosine of sphingosine kinase in *A. thaliana* is 3 μM (10), about 50 times higher than the concentration of sphingadienine in Os3BGlu6-deficient rice mutants (about 0.06 μM, Fig. 6*C*). The enzymatic activity tends to correlate with the substrate concentration if the *K*_m_ value of the enzyme is much higher than the substrate concentration. Thus, these results indicate that the decrease in the level of sphingadienine-1-phosphate in each Os3BGlu6-deficient rice mutant is due to the decrease in the level of sphingadienine, a substrate of sphingosine kinase.

### Distribution of GH1 GCase in plants

**Figure 7:**
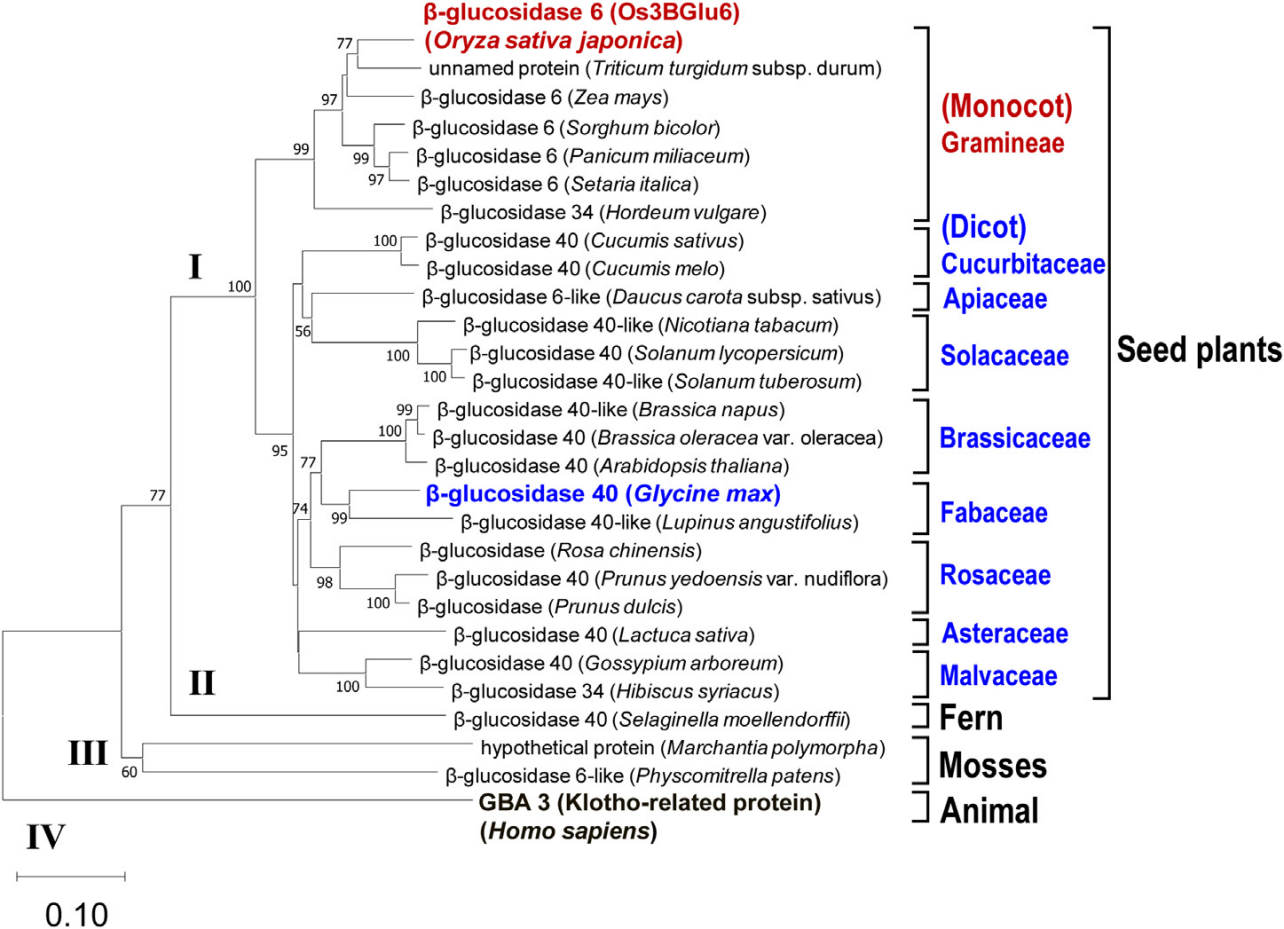
**Phylogenetic tree of amino acid sequences of Os3BGlu6 and the most similar protein in various plant and animal species.** The protein most similar to Os3BGlu6 was selected using a BLASTp analysis of the NCBI database (https://blast.ncbi.nlm.nih.gov/Blast.cgi). Multiple sequences were aligned using multiple sequence comparison by log-expectation (MUSCLE) algorithm (44) implemented in MEGA X (45). The phylogenetic tree was constructed using MEGA X. Bootstrap values (>50%) at the nodes are based on 1000 replicates.

**Figure 8:**
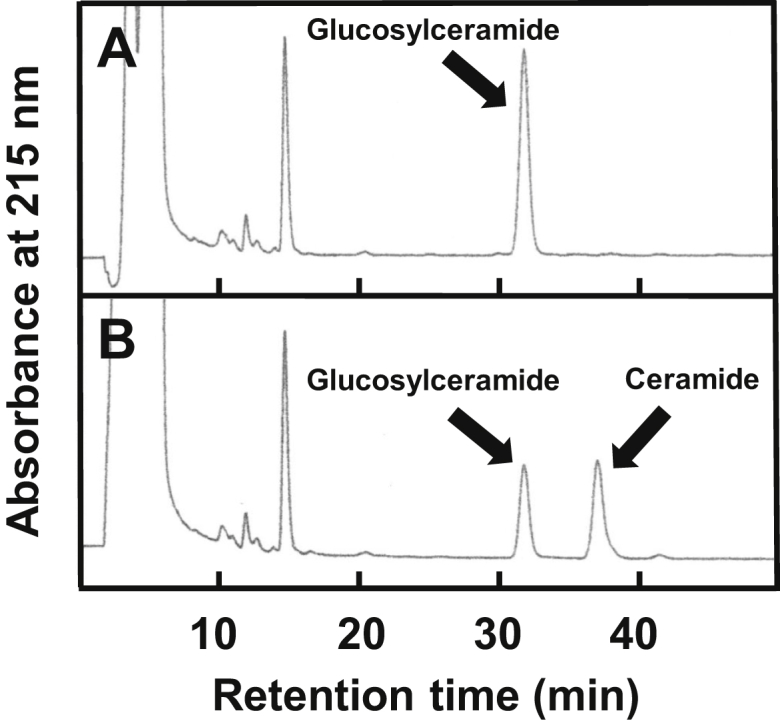
**GCase activity of soybean β-glucosidase 40**. HPLC chromatograms (*A*) before and (*B*) after incubation of a fungal glucosylceramide [d19:2(4*E*,8*E*,9Me)-C18h:1-GluCer] with recombinant soybean β-glucosidase 40 protein extracts expressed in *E. coli*.

Finally, we examined whether GH1 GCase is widely distributed in the other plants. The protein most similar to Os3BGlu6 was selected from each of plant and animal species using a BLASTp search of the NCBI database (https://blast.ncbi.nlm.nih.gov/Blast.cgi). The phylogenetic tree of amino acid sequences from Os3BGlu6 and those from the most similar protein in each plant and animal clearly grouped into four clusters (I–IV), belonging to seed plants (I), ferns (II), mosses (III), and animals (IV) (Fig. 7). The conserved GH1 residues Q31, H132, N177, E178, E394, E451, and W452 found in Os3BGlu6 were present in the all the amino acid sequences, suggesting that these proteins belong to the GH1 group. Among them, the protein that has been already identified as GCase was Klotho-related protein (human GBA3) only (30), which shared 35.8% sequence identity with Os3BGlu6. However, the *K*_cat_/*K*_m_ value for synthetic glucosylceramide (C6-NBD-GluCer) of Os3BGlu6 was 1.97 ± 0.12 s^−1^/μM (Table 1), about 75 times higher than that of Klotho-related protein (0.0262 ± 0.0005 s^−1^/μM) (30). Furthermore, the *K*_cat_/*K*_m_ value for animal glucosylceramide [d18:1(4*E*)-C12:0-GluCer] of Os3BGlu6 was 2.64 ± 0.19 s^−1^/μM (Table 1), about 5280 times higher than that for animal glucosylceramide [d18:1(4*E*)-C18:0-GluCer] of Klotho-related protein (0.0005 ± 0.0000 s^−1^/μM) (30), indicating that Os3BGlu6 is a novel GCase, different from Klotho-related protein. BLASTp analysis showed that Os3BGlu6 shared 77–88% sequence identity with its most similar protein (β-glucosidase 6; Fig. 7) in each monocot plant and shared 63–75% sequence identity with its most similar protein (β-glucosidase 40; Fig. 7) in each dicot plant, suggesting that β-glucosidase 6 is a GH1 GCase. Therefore, to confirm whether β-glucosidase 40 from dicot plants is also a GH1 GCase, the β-glucosidase 40 gene was cloned from the dicot *Glycine max* L. cv. Enrei, and the gene was expressed in *E. coli*. Glucosylceramide was efficiently converted to ceramide by the recombinant protein expressed in *E. coli* (Fig. 8, *A* and *B*), suggesting that β-glucosidase 40 from *G*.*max* L. cv. Enrei has GCase activity. It seems likely that β-glucosidase 40 and β-glucosidase 6 belonging to cluster I are also a GH1 GCase.

**Figure 9:**
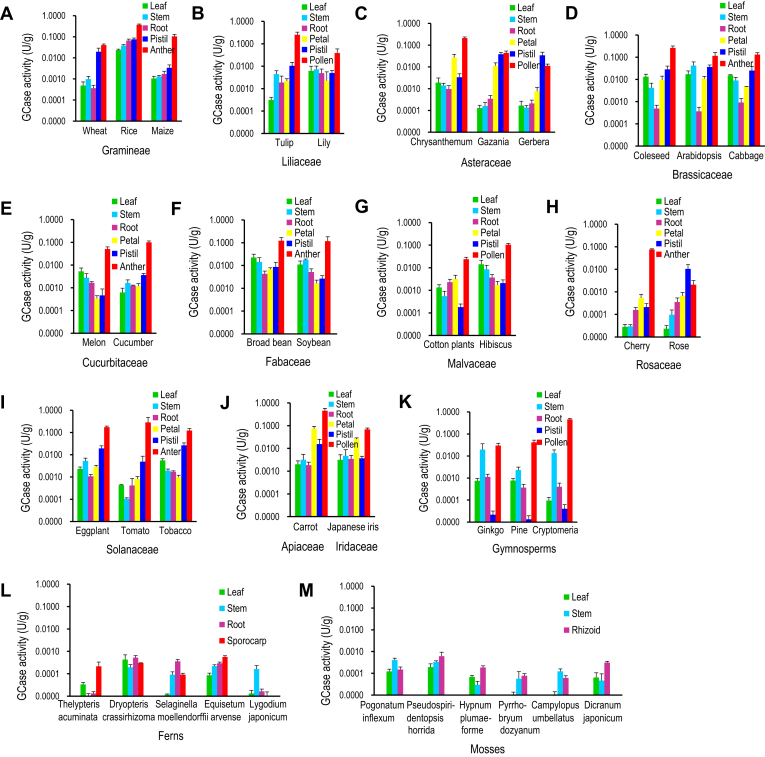
**GCase activity in various plant species.** GCase activity in organs of angiosperm (*A–J*), gymnosperm (*K*), fern (*L*), and moss (*M*) species. GCase activity is expressed as units (U) per fresh weight (g) of plant material (*n* = 3).

Next, we examined whether seed plants actually produce GH1 GCase by measuring the GCase activity in species from a broad range of plant families. GCase activity was detected in the extracts from all seed plants tested (species of Gramineae, Liliaceae, Asteraceae, Brassicaceae, Cucurbitaceae, Fabaceae, Malvaceae, Rosaceae, Solanaceae, Apiaceae, Iridaceae, Ginkgoaceae, Pinaceae, and Taxodiaceae; Fig. 9, *A*–*K*). Furthermore, the activity in pollen or anthers from all species was 10–10,000 times higher than in other organs, the same as in rice. On the other hand, activity was barely detected or very low in extracts from ferns (Pteridophyta) and mosses (Bryophyta) (Fig. 9, *L* and *M*).

Although the GCase activity in the tested seed plants could be due to a GCase other than GH1 GCase, GCase activity of GH1 GCase-deficient rice mutants was completely absent relative to that of the wild type (Fig. 2*A*). Thus, we can reasonably assume that almost the GCase activity in the tested species is due to GH1 GCase and that seed plants produce high levels of GH1 GCase.

## Discussion

Gaucher's disease, the most prevalent lysosomal storage disorder, is caused by mutations in the gene encoding a lysosomal GCase, leading to glucosylceramide accumulation in the liver, spleen, and bone marrow and to severe complications such as thrombocytopenia and skeletal deterioration (1, 2). Therefore, a recombinant human GCase has been developed as an enzyme replacement therapy (3, 4). Although animal GCases have been extensively studied and are important for regulating physiology, the physiological functions of GCases in plants remain unclear. Our findings are the first to show that a novel GH1 GCase is widely distributed in seed plants and that it regulates the level of ceramides containing sphingadienine, influencing the regulation of sphingadienine-1-phosphate levels and subsequent improvement of drought tolerance *via* stomatal closure in rice as shown in Figure 4.

In the experiments on the regulation of ceramide levels, the amounts of ceramides containing sphingadienine in anthers of Os3BGlu6-deficient rice mutants were 20–60 times lower than in the wild type, but the amounts of ceramides containing 4-hydroxysphingenine in the anthers were only 2–3 times lower than in the wild type (Fig. 2*D*). Since the LCBs of glycosylinositol phosphoceramide (GIPC) are mainly composed of 4-hydroxysphinganine, 4-hydroxysphingenine, or sphingenine in rice (25), it is possible that the ceramides containing 4-hydroxysphingenine in the anthers were synthesized from GIPC as well as from glucosylceramide as shown in Figure 4. Considering these facts and our enzymatic results that GCase activity of Os3BGlu6 for glucosylceramides containing 4-hydroxysphingenine was lower than for glucosylceramides containing sphingadienine (Table 2), it seems plausible that the level of ceramides containing 4-hydroxysphingenine is regulated by GIPC-related enzymes as well as by Os3BGlu6 (Fig. 4).

In the experiments on the regulation of LCBP levels, among LCBPs, the amounts of sphingadienine-1-phosphate in Os3BGlu6-deficient rice mutants were significantly lower than in the wild type, but neither the amounts of phytosphingosine-1-phosphate nor sphingosine-1-phosphate differed between the mutants and the wild type (Fig. 5, *B* and *C*). Rice glucosylceramides were previously found to be mainly composed of sphingadienine but rice GIPCs were mainly composed of 4-hydroxysphinganine, 4-hydroxysphingenine, or sphingenine (25). Thus, this result is reasonable because sphingadienine-1-phosphate is synthesized from ceramides containing sphingadienine, which are regulated by Os3BGlu6, but phytosphingosine-1-phosphate or sphingosine-1-phosphate might be mainly synthesized from ceramides containing 4-hydroxysphinganine or sphingenine derived from GIPCs (Fig. 4).

In the experiments on the induction of a defense response, the amounts of phytoalexins induced in the leaves of Os3BGlu6-deficient rice mutants with fungal glucosylceramide elicitor treatment were almost the same as those of the wild type (Fig. 3*B*), suggesting that the ceramide generated from the fungal glucosylceramide by Os3BGlu6 does not seem likely to function as an elicitor of defense response such as phytoalexin induction. Since stomata are located in the leaf and stem surface in plants, they are the main entry point for pathogens (31). Melotto *et al.* (32) revealed that stomata passively function as a barrier against invasion of pathogens. For example, in *A. thaliana*, stomata rapidly closed upon contact with pathogenic bacteria such as *Pseudomonas syringae* pv. tomato. Chitosan isolated from cell walls of pathogenic fungi also induced stomatal closure in *A. thaliana* (33). In the present study, we showed that Os3BGlu6 increased the level of sphingadienine-1-phosphate in rice (Fig. 5, *B* and *C*). If the ceramide generated from the fungal glucosylceramide by Os3BGlu6 is metabolized to methylsphingadienine-1-phosphate, leading to stomatal closure as shown in Figure 4, the ceramide might function as an elicitor of a defense response such as stomatal closure rather than phytoalexin induction in rice.

Wang *et al.* (7) reported that the amounts of Os3BGlu6 from a rice plant overexpressing *Os3BGlu6* gene (OE-5 plant) were much higher than those from the wild type in western blotting analysis and that the hydrolysis of glucose-conjugated abscisic acid (ABA-GE) to ABA by the crude enzyme extracted from the OE-5 plant was 22.6% higher than from the wild type. From these results, they proposed that Os3BGlu6 facilitates the hydrolysis of ABA-GE to ABA, thereby increasing the cellular ABA level in rice (7). However, our present study demonstrated that Os3BGlu6 is a GH1 GCase and specifically recognizes the glucosylceramide structure. High amounts of Os3BGlu6 produced from the OE-5 plant might catalyze the hydrolysis of a small amount of ABA-GE to ABA, but we consider that the contribution of this ABA-GE hydrolase activity to ABA synthesis is low in the wild type because low amounts of Os3BGlu6 produced in the wild type likely do not hydrolyze ABA-GE to ABA.

**Figure 10:**
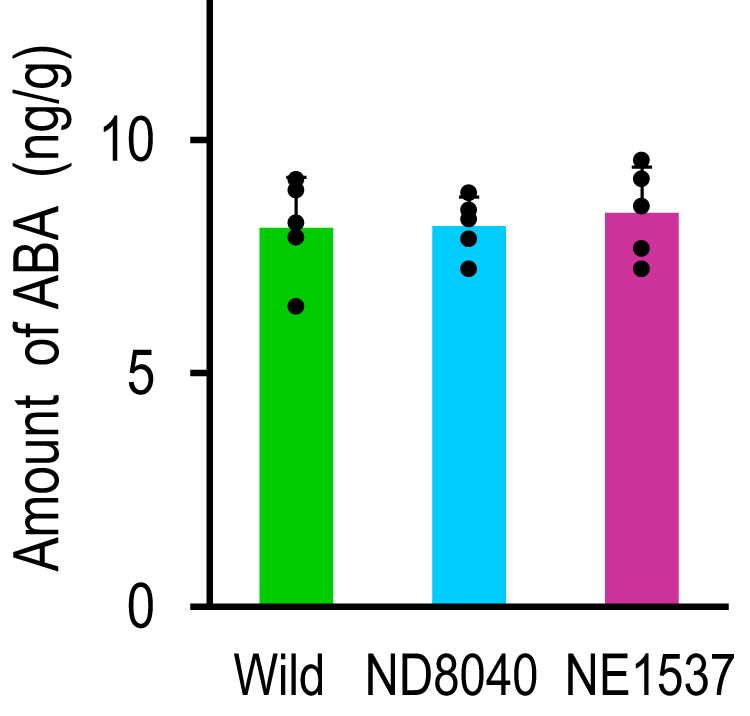
**Amount ofABAin Os3BGlu6-deficient rice mutants and the wild type.** Amount of ABA in leaves of Os3BGlu6-deficient rice mutants NE1537 and ND8040 and the wild type (ng/g fresh weight of plant material). *Asterisks* indicate a significant difference in the amount of ABA in leaves of either mutant compared to that of the wild type in Student's *t* test (∗*p* < 0.05, ∗∗*p* < 0.01, *n* = 5). ABA, abscisic acid.

When plants lose water, they elevate cellular ABA levels, then ABA activates sphingosine kinase activity, leading to an increase in the level of LCBPs that regulate stomatal closure as shown in Figure 4 (10, 11). Thus, if Os3BGlu6 elevates cellular ABA levels in rice as described (7), it should activate sphingosine kinase, leading to an increase in the level of LCBPs. However, in the present study, we showed that sphingosine kinase activity and the levels of LCBPs such as sphingosine-1-phosphate and phytosphingosine-1-phosphate did not differ significantly between the Os3BGlu6-deficient rice mutants and the wild type (Figure 5, Figure 6*B*). These results indicate that Os3BGlu6 might not increase the ABA level in rice. Therefore, to examine whether Os3BGlu6, indeed increases the ABA level in rice, we measured the amount of ABA in the Os3BGlu6-deficient rice mutants. As shown in Figure 10, the amounts of ABA did not differ significantly between the Os3BGlu6-deficient rice mutants (NE1537 and ND8040) and the wild type. This result indicates that Os3BGlu6 regulates sphingadienine-1-phosphate levels in the ABA-independent pathway, leading to stomatal closure in rice as shown in Figure 4.

LCBPs are known to be signaling molecules for improving drought tolerance in plants (8, 9, 10, 11). In the present study, the levels of LCBPs, especially sphingadienine-1-phosphate, correlated with GCase activity in each rice organ (Figs. 2*B* and 5*A*), suggesting that high GCase activity leads to a high level of sphingadienine-1-phosphate, thereby improving drought tolerance in plants. Thus, we speculate that seed plants with high GCase activity produce a high level of sphingadienine-1-phosphate, resulting in resistance to drought, whereas ferns and mosses with low GCase activity produce only a low level of sphingadienine-1-phosphate, making them sensitive to drought. Seed plants might have acquired the ability to synthesize a GH1 GCase with high activity for drought tolerance, but ferns and mosses that grow only in a humid environment might not need a GH1 GCase with such high activity.

In animals, LCBPs are signaling molecules involved in cell growth and apoptosis suppression (34, 35). The level of LCBP is known to be regulated by LCB kinase, LCBP phosphatase, and LCBP lyase (8, 36), but GCase has not been reported before the present study to regulate LCBPs in plants and animals. Thus, our discovery of GH1 GCase in plants opens a new avenue for research on LCBP-signaling involved in stomatal closure, disease resistance, cell growth, and apoptosis suppression in plants and animals.

## Experimental procedures

### Materials

Fungal glucosylceramides, (4*E*,8*E*)-*N*-d-2′-hydroxy-(*E*)-3′-hexadecenoyl-1-*O*-β-d-glucopyranosyl-9-methyl-4,8-sphingadienine [d19:2(4*E*,8*E*,9Me)-C16h:1-GluCer]; (4*E*,8*E*)-*N*-d-2′-hydroxy-(*E*)-3′-octadecenoyl-1-*O*-β-d-glucopyranosyl-9-methyl-4,8-sphingadienine [d19:2(4*E*,8*E*,9Me)-C18h:1-GluCer] were isolated from the fungus *P. oryzae* as described (18). A fungal glucosylceramide, (4*E*,8*E*)-*N*-d-2′-hydroxypalmitoyl-1-*O*-β-d-glucopyranosyl-9-methyl-4,8-sphingadienine [d19:2(4*E*,8*E*,9Me)-C16h:0-GluCer], plant glucosylceramides, and sphingadienine were purchased from Nagara Science. Animal sphingolipids were purchased from Avanti Polar Lipids. Sphingosine-1-phosphate and phytosphingosine-1-phosphate were purchased from Fujifilm Wako Pure Chemical. Standard ceramides for LC-ESI-MS/MS were enzymatically synthesized from the standard glucosylceramides by recombinant human GCase (Imiglucerase, EC 3.2.1.45, GH family 30) as described previously (26). Standard sphingadienine-1-phosphate for LC-ESI-MS/MS was enzymatically synthesized from the standard sphingadienine by recombinant human sphingosine kinase 1 (Cayman Chemical). For the conversion of sphingadienine, reaction mixtures (100 µL) with 10 μl of recombinant human sphingosine kinase 1, 1 μM sphingadienine, 0.5 mM adenosine triphosphate (ATP), 50 mM NaCl, 10 mM MgCl_2_, 0.1% Triton X-100, and 100 mM Tris-HCl (pH 7.4) were incubated for 8 h at 37 °C. The reaction was diluted with methanol to 0.1 μM sphingadienine-1-phosphate.

Nursery plants, seeds, and bulbs were purchased from Takii Seed, Sakata Seed, Tsurusin Seedling, and Japan Agricultural Cooperatives. Seeds of *A. thaliana* and *Nicotiana tabacum* were kindly provided by Dr Hiroyuki Anzai (Ibaraki University), Ferns were kindly provided by Drs. Takao Yokota and Masashi Asahina (Teikyo University). Mosses were purchased from Mossfarm.

Wheat (*Triticum aestivum*), rice (*Oryza sativa* cv. Nipponbare), maize (*Zea mays* cv. Iltis), tulip (*Tulipa gesneriana*), lily (*Lilium concolor*), lupine (*Lupinus hirsutus*), broad bean (*Vicia faba*), soybean (*G*. *max* cv. Enrei), coleseed (*Brassica napus*), *A. thaliana*, radish (*Raphanus sativus* var. longipinnatus), cabbage (*Brassica oleracea* var. capitata), eggplant (*Solanum melongena*), tomato (*Solanum lycopersicum*), tobacco (*N. tabacum*), bitter gourd (*Momordica charantia* var. pavel), melon (*Cucumis melo* var. reticulatus), cucumber (*Cucumis sativus*), rose (Rosa sp.), lettuce (*Lactuca sativa* var. capitata), gazania (*Gazania rigens*), chrysanthemum (*Coreopsis basalis*), gerbera (*Gerbera jamesinoii*), cotton (*Gossypium arboreum* var. obtusifolium), hibiscus (*Hibiscus rosa-sinensis*), carrot (*Daucus carota* subsp. sativus), and Japanese iris (*Iris ensata* var. ensata) were grown in a greenhouse. After cultivation, their leaves, stems, roots, petals, pistils, anthers, and pollens were collected. The samples from wisteria (*Wisteria floribunda*), cherry (*Prunus xyedoensis*), ginkgo (*Ginkgo biloba*), pine (*Pinus thunbergii*), and cryptomeria (*Cryptomeria japonica*) were collected at parks in Shiki or Sakado city (Saitama Japan). We could not collect cryptomeria and ginkgo pollen, so they were purchased from Fujifilm Wako Pure Chemical.

Os3BGlu6-deficient rice mutants NE1537 and ND8040, which are inserted retrotransposon *Tos17* in the region of *Os3BGlu6* gene of rice (*Oryza sativa* cv. Nipponbare), were provided from Rice Genome Resource Center, Institute of Crop Science, NARO, Ibaraki Japan.

### Purification and identification of compound 1 liberated from glucosylceramide by rice GCase

Ten grams of rice leaves was homogenized in 100 ml of Buffer L at 4 °C. The mixture was centrifuged at 36,000*g* for 60 min, and the supernatant was dialyzed against Buffer H. One milliliter of 10 mM glucosylceramide [d19:2(4*E*,8*E*,9Me)-C18h:1-GluCer] dissolved in ethanol was added to 50 ml of the dialyzed solution and 49 ml of 0.4% sodium cholate, then the mixture was incubated for 1 h at 37 °C. The reaction mixture was then adjusted to pH 11.5 with Na_2_CO_3_ and NaOH solutions and mixed with ethyl acetate for extraction. The ethyl acetate extract was evaporated, then fractionated by HPLC using a TSKgel ODS-120T column (4.6 mm i.d. × 30 cm; Tosoh) that was eluted with 81% ethanol at a flow rate of 1.0 ml/min at 50 °C. The eluate was monitored with an ultraviolet detector at 215 nm. A new peak (compound 1) liberated from the glucosylceramide was collected (Fig. 1*C*) and confirmed as the ceramide [d19:2(4*E*,8*E*,9Me)-C18h:1-Cer] by LC-ESI-MS/MS (Precursor ion *m*/*z*: 592.6 [M+H]^+^, product ion m/z: 276.2).

### Analytical procedures

#### Analysis of LCBPs by LC-ESI-MS/MS

Rice plants were grown in pots in a greenhouse until the eight-leaf stage. Before chemical analysis, they were soaked once in water until soil was saturated, then grown without watering for 24 h, then young leaves, stems, and roots (0.1–1.0 g) were collected. At the heading stage, pistils and anthers (0.002–0.05 g) were collected and extracted with methanol. The methanol extract was then evaporated to a final volume of 0.32 ml, then 0.04 ml of 0.2 M HCl and 0.04 ml of isopropanol were added to the sample, which was then mixed and centrifuged twice at 20,000*g* for 30 min at 4 °C. The supernatant (8 μl) was subjected to LC-ESI-MS/MS equipped with a ZORBAX Eclipse XDB-C18 column (2.1 mm i.d. × 5 cm, Agilent) and eluted with 90% solvent A (60% methanol +39.8% H_2_O + 0.2% formic acid) plus 10% solvent B (60% methanol + 39.8% isopropanol + 0.2% formic acid) for 5 min at 0.2 ml/min at 40 °C, followed by a linear gradient of 90% solvent A plus 10% solvent B to 100% solvent B for 15 min at 0.2 ml/min at 40 °C. LCBPs such as sphingosine-1-phosphate, phytosphingosine-1-phosphate, and sphingadienine-1-phosphate, which are available as reference standards, were analyzed in positive ion mode with nitrogen as the collision gas and quantified by multiple reaction monitoring (MRM) as described previously (27, 28, 29).

The ESI source parameters were sheath gas flow rate, 12 l/min; sheath gas temperature, 300 °C; nebulizer pressure, 30 psi; capillary voltage, 4000 V; fragmentor voltage, 105 V; collision energy, 12 eV for sphingosine-1-phosphate, 10 eV for phytosphingosine-1-phosphate, and 8 eV for sphingadienine-1-phosphate; precursor ion [M+H]^+^, *m*/*z* 380.1 for sphingosine-1-phosphate, *m*/*z* 398.2 for phytosphingosine-1-phosphate, and *m*/*z* 378.1 for sphingadienine-1-phosphate; product ion, *m*/*z* 264.2 for sphingosine-1-phosphate, *m*/*z* 300.2 for phytosphingosine-1-phosphate, and *m*/*z* 262.2 for sphingadienine-1-phosphate.

#### Analysis of ceramides by LC-ESI-MS/MS

Ceramides were measured essentially as previously described (26). At the eight-leaf stage, young leaves, stems, and roots (0.05–0.5 g) were collected and at the heading stage, pistils and anthers (0.002–0.05 g) were collected and extracted with ethanol. The extract was evaporated, then dissolved in 0.80 ml of ethanol before adding 0.20 ml of 0.2 M HCl. The solution was mixed and centrifuged twice at 20,000*g* for 30 min at 4 °C. The supernatant was subjected to LC-ESI-MS/MS equipped with a TSKgel ODS-120A column (2.1 mm i.d. × 25 cm, Tosoh) and eluted with a linear gradient of 10% solvent A (H_2_O + 0.1% formic acid) plus 90% solvent B (methanol + 0.1% formic acid) to 2% solvent A plus 98% solvent B for 30 min at 0.2 ml/min at 40 °C, followed by 2% solvent A plus 98% solvent B for 20 min at 0.2 ml/min at 40 °C. Main rice ceramides were analyzed in positive ion mode with nitrogen as the collision gas and quantified by MRM as described previously (26).

#### Analysis of sphingadienine by LC-ESI-MS/MS

At the eight-leaf stage, young leaves (0.05–0.5 g) were collected and extracted with ethanol. The extract was evaporated, then dissolved in 0.80 ml of methanol before adding 0.20 ml of H_2_O. The solution was mixed and centrifuged twice at 20,000*g* for 30 min at 4 °C. The supernatant (3 μl) was subjected to LC-ESI-MS/MS to measure the amount of sphingadienine as described in the LCBP analysis section. Sphingadienine was analyzed in positive ion mode with nitrogen as the collision gas and quantified by MRM as described previously (27).

The ESI source parameters were sheath gas flow rate, 12 l/min; sheath gas temperature, 300 °C; nebulizer pressure, 30 psi; capillary voltage, 4000 V; fragmentor voltage, 80 V; collision energy, 6 eV; precursor ion [M+H]^+^, *m*/*z* 298.3; product ion, *m*/*z* 280.3.

#### Analysis of ABA by LC-ESI-MS/MS

ABA was measured essentially as previously described (37). At the eight-leaf stage, young leaves (0.4–2.0 g) were collected and extracted with methanol containing 1% acetic acid. The extract was evaporated to a final volume of 0.8 mL, then 0.2 mL of 0.1 M HCl was added to the sample, which was then mixed and centrifuged twice at 20,000*g* for 30 min at 4 °C. The supernatant (5 μl) was subjected to LC-ESI-MS/MS equipped with a ZORBAX Eclipse XDB-C18 column (2.1 mm i.d. × 5 cm, Agilent) and eluted with a linear gradient of 97% solvent A (H_2_O + 0.05% acetic acid) plus 3% solvent B (acetonitrile + 0.05% acetic acid) to 30% solvent A plus 70% solvent B for 15 min at 0.2 ml/min at 40 °C, followed by 2% solvent A plus 98% solvent B for 5 min at 0.3 ml/min at 40 °C. ABA was analyzed in negative ion mode with nitrogen as the collision gas and quantified by MRM as described previously (37).

The ESI source parameters were sheath gas flow rate, 12 l/min; sheath gas temperature, 350 °C; nebulizer pressure, 40 psi; capillary voltage, 3000 V; fragmentor voltage, 150 V; collision energy, 4 eV; precursor ion [M-H]^−^, *m*/*z* 263.2; product ion, *m*/*z* 153.1.

### Ceramidase assay

Ceramidase assay was measured essentially as previously described (38). At the eight-leaf stage, young leaves (0.5–1.0 g) were homogenized with 20 ml of Buffer O at 4 °C. The mixture was centrifuged at 20,000*g* for 20 min at 4 °C, and the supernatant was collected as the enzyme solution. A ceramide [d18:2(4*E*,8*Z*)-C16h:0-Cer] was dissolved in 0.2% Triton X-100 solution using sonication and mixing at 65 °C. Reaction mixtures with the enzyme solution and 25 μM of d18:2(4*E*,8*Z*)-C16h:0-Cer in 0.2 ml of Buffer N were incubated for 30 min at 37 °C. The reaction was stopped by adding 0.8 ml of 100% methanol and centrifuged at 20,000*g* for 30 min. The supernatant (3 μl) was subjected to LC-ESI-MS/MS to measure the amount of sphingadienine as described in the sphingadienine analysis section.

### Sphingosine kinase assay

Sphingosine kinase assay was measured essentially as previously described (10). At the eight-leaf stage, young leaves (0.2–1.0 g) were homogenized with 20 ml of Buffer P at 4 °C. The mixture was centrifuged at 20,000*g* for 20 min at 4 °C, and the supernatant was collected as the enzyme solution. Sphingadienine was dissolved in 0.2% Triton X-100 solution using sonication and mixing at 55 °C. Reaction mixtures with the enzyme solution and 50 μM of sphingadienine in 0.1 ml of Buffer Q were incubated for 60 min at 37 °C. The reaction was stopped by adding 0.1 ml of 0.2 M HCl and 0.8 ml of methanol, then centrifuged at 20,000*g* for 30 min. The supernatant (8 μl) was subjected to LC-ESI-MS/MS to measure the amount of sphingadienine-1-phosphate as described in the LCBP analysis section.

### GCase assay

#### Buffers

The buffers used were as follows; Buffer A, 20 mM sodium acetate and 0.05% sodium cholate (pH 5.5); Buffer B, 20 mM sodium acetate and 0.05% sodium cholate (pH 6.0); Buffer C, Buffer B plus 0.1% Triton X-100 (pH 6.0); Buffer D, Buffer C plus 0.1 M NaCl (pH 6.0); Buffer E, Buffer B plus 0.1% Triton X-100 (pH 5.5); Buffer F, Buffer C plus 0.08 M NaCl (pH 5.8); Buffer G, Buffer C plus 0.1 M NaCl (pH 5.8); Buffer H, 40 mM sodium acetate, 0.025% sodium cholate, and 0.05% Tween 20 (pH 5.5); Buffer I, 50 mM sodium acetate and 0.05% sodium cholate (pH 6.0); Buffer J, Buffer I plus 0.1% Tween 20 (pH 5.5); Buffer K, Buffer I plus 0.3% Triton X-100 (pH 5.0); Buffer L, Buffer I plus 0.3% Triton X-100 (pH 5.5); Buffer M, Buffer I plus 0.2% Triton X-100 (pH 5.0); Buffer N, 50 mM potassium phosphate, 5 mM MgCl_2_, and 0.2% Triton X-100 (pH 6.0); Buffer O, Buffer N plus 1 mM dithiothreitol (DTT) (pH 6.0); Buffer P, 100 mM Tris-HCl, 10 mM MgCl_2_, 1 mM DTT and 0.2% Triton X-100 (pH 7.4); Buffer Q, 100 mM Tris-HCl, 10 mM MgCl_2_, 1 mM ATP, and 0.2% Triton X-100 (pH 7.4).

#### Enzyme assays

Certain glucosylceramides were not completely soluble at high concentrations, so the substrate specificity experiment was carried out using 12.5 μM of substrate, a concentration at which all substrates were soluble. Each glucosylceramide was dissolved in 0.1% Tween 20 solution using sonication and mixing at 65 °C. For stabilizing GCase, the enzyme solution was preincubated with Buffer L for 30 min at 37 °C. Reaction mixtures with the stabilized purified rice GCase and 12.5 μM of each sphingolipid substrate in 0.2 ml of Buffer J were incubated for 15 min at 37 °C. The reaction was stopped by adding 0.8 ml of 100% ethanol and centrifuged at 20,000*g* for 30 min. Each supernatant (0.3 ml) was subjected to HPLC on a TSKgel Octyl-80Ts column (4.6 mm i.d. × 30 cm; Tosoh) eluted with 87% ethanol at 1.0 ml/min at 50 °C for the analysis of d18:1(4*E*)-C18:0-Cer and a TSKgel ODS-120T column (4.6 mm i.d. × 30 cm; Tosoh) eluted with 82% acetonitrile at 1.0 ml/min at 50 °C for the analysis of d18:1(4*E*)-C8:0-Cer, 85% acetonitrile for the analysis of d18:1(4*E*)-C12:0-Cer, and 85–89% ethanol for the analysis of the other ceramides. The effluent was monitored with an ultraviolet detector at 215 nm. The ceramide concentration in each reaction solution was determined using a standard curve based on ceramide hydrolyzed from standard glucosylceramide by imiglucerase (26). One unit (U) of activity was defined as the amount of enzyme releasing 1 μmole of ceramide per min.

#### Enzyme kinetics

Glucosylceramides containing a long-chain fatty acid were not completely soluble at concentrations above 15 μM, so the kinetic parameters of glucosylceramides containing the fatty acid of C16 or lower chain length were measured (Table 1). Various substrate concentrations were used to test reaction velocity and kinetic parameters of the reactions determined from a Hanes–Woolf plot. Low levels of plant ceramides were difficult to detect at 215 nm with an ultraviolet detector, so each plant ceramide sample was subjected to LC-ESI-MS/MS. Respective ceramides were quantified by MRM as described previously (26). The results were expressed as the mean ± SD of five experiments.

#### Measurement of protein concentration

The addition of more than 0.1% Triton X-100 and 0.05% sodium cholate to rice GCase was essential for stabilizing the enzyme. However, protein concentrations in enzyme solutions containing Triton X-100 are not correctly measured by the Protein Assay Kit (Bio-Rad), because Triton X-100 in the solution also causes the blue color change that proteins cause. Therefore, the protein concentration of rice GCase was measured by HPLC analysis on a TSKgel Octyl-80Ts column (4.6 mm i.d. × 15 cm; Tosoh) eluted with a linear gradient of 0 to 60% acetonitrile in 0.05% trifluoroacetic acid (TFA) at 0.8 ml/min. The peak was monitored with an ultraviolet detector at 280 nm. The protein concentration of rice GCase was determined by comparison with the peak area for recombinant human GCase (Imiglucerase), for which the concentration was previously determined by the Protein Assay Kit (Bio-Rad) using bovine serum albumin as the standard (39).

#### GCase activity of the extracts from plants

Leaves, stems, roots, petals, pistils, anthers, pollens, sporocarps, and rhizoids from plants were collected and cut into pieces. Each sample (0.01–2.0 g) was homogenized with 20 ml of Buffer K at 4 °C. The mixture was centrifuged at 20,000*g* for 20 min, and the supernatant was collected as the enzyme solution. For stabilizing GCase, the extracted enzyme solution was preincubated with Buffer K for 30 min at 37 °C. For the activity assay, reaction mixtures with the stabilized enzyme and a glucosylceramide [d19:2(4*E*,8*E*,9Me)-C16h:0-GluCer] in 0.2 ml of Buffer M were incubated for 60 min at 45 °C. After incubation, the liberated ceramide was measured by HPLC on a TSKgel ODS-120T column (4.6 mm i.d. × 30 cm; Tosoh) eluted with 85% ethanol as described above. GCase activity was defined as the number of units per fresh weight (g) of the sample. The results were expressed as the mean ± SD of three experiments.

### Enzyme purification of GCase from rice leaves

#### Step 1: extraction of GCase from rice leaves with a nonionic surfactant

Rice plants (*Oryza sativa* cv. Nipponbare) were grown in a greenhouse, and at the ten-leaf stage, the young leaves were collected and cut into pieces; 110 g of the sample was then homogenized with 1.3 L of Buffer B at 4 °C. The mixture was centrifuged at 18,800*g* for 60 min. The supernatant was discarded, and the pellet and 1.3 L of Buffer B were mixed for 10 min at 4 °C. The mixture was again centrifuged at 18,800*g* for 60 min at 4 °C, the supernatant was discarded, and 755 ml of Buffer B plus 0.06% Triton X-100 was added to 145 g of the pellet and mixed for 30 min at 25 °C. The GCase solution that was extracted with Triton X-100 was centrifuged at 36,000*g* for 60 min. The supernatant was collected and filtered through a membrane filter (0.45 μm pore size). The filtrate (800 ml) was concentrated to 30 ml using an Amicon ultrafiltration 30K (Merck Millipore) and then ultrafiltered five times with Buffer I. The entire protocol was done three times; thus, using 330 g of rice leaves in total, 90 ml of the final filtrate was obtained.

#### Step 2: purification of GCase using anion exchange chromatography

Since the addition of more than 0.1% Triton X-100 and 0.05% sodium cholate to rice GCase was essential for stabilizing the enzyme, the concentration of Triton X-100 needed to be monitored. The Triton X-100 solution becomes blue after the addition of the reagent from the Protein Assay Kit; therefore, the concentration of Triton X-100 was measured using the Protein Assay Kit in the same way as for proteins. The 15 ml of the sample obtained in Step 1 was diluted to be finally 50 ml of 0.25% Triton X-100, 0.05% sodium cholate, and 35 mM sodium acetate (pH 6.0), then 50 ml of the diluted sample was subjected to FPLC (GE Healthcare) on a HiTrap Q HP column (5 ml × 3, GE Healthcare) equilibrated with Buffer C. After washing with three-bed volumes of Buffer C, the column was eluted with a linear gradient from Buffer C to Buffer D at 3 ml/min. This process was done six times, and the 90 ml of the sample obtained in Step 1 was purified. The active fractions were pooled and concentrated to 10 ml using an Amicon ultrafiltration 30K (Merck Millipore), then ultrafiltered seven times with Buffer I.

#### Step 3: purification of GCase using cation exchange chromatography

Part of the sample (2.5 ml) obtained in Step 2 was diluted to be finally 30 ml of 0.2% Triton X-100, 0.05% sodium cholate, and 20 mM sodium acetate (pH 5.5), then 30 ml of the diluted sample was subjected to FPLC on a HiTrap SP HP column (5 ml × 2, GE Healthcare) equilibrated with Buffer E. After washing with six bed volumes of Buffer E, the sample was eluted with a linear gradient from Buffer E to Buffer F at 3 ml/min. This process was done four times to purify 10 ml of the sample obtained in Step 2. The active fractions were pooled and concentrated to 4 ml using an Amicon ultrafiltration 30K (Merck Millipore) and then ultrafiltered five times with Buffer I.

### Identification of the purified rice GCase by MALDI-TOF MS

#### SDS-PAGE

SDS-PAGE was performed using a ready-made 10% gel (e-PAGEL E-R10L, ATTO) (40). After electrophoresis, the gel was stained using the Silver Stain MS Kit (Fujifilm Wako Pure Chemical). SDS-PAGE low-molecular-weight standards were used as the molecular mass markers (Bio-Rad).

#### In-gel protein digestion

The 62-kDa protein band on SDS-PAGE gel was enzymatically digested in-gel using modified porcine trypsin (Promega) (41). The resultant gel pieces were washed with 50% acetonitrile and vacuum dried, then rehydrated with trypsin solution (8–10 ng/μl 50 mM ammonium bicarbonate, pH 8.7), and incubated for 8–10 h at 37 °C.

#### MALDI-TOF MS

For identification of components of the 62-kDa band on SDS-PAGE (Fig. 1*F*) by peptide mass fingerprinting (PMF) with MALDI-TOF MS, the in-gel-digested protein sample was mixed with α-cyano-4-hydroxycinnamic acid in 50% acetonitrile and 0.1% TFA and subjected to MALDI-TOF MS (Microflex LRF 20, Bruker Daltonics) as described (42). Spectra were collected from 300 shots per spectrum over *m*/*z* range 600 to 3000 and calibrated by two-point internal calibration using trypsin auto-digestion peaks (*m*/*z* 842.5099, 2211.1046). Peak list was generated using Flex Analysis 3.0. Threshold used for peak-picking was as follows: 500 for minimum resolution of monoisotopic mass, 5 for S/N. The search program Mascot (43) developed by Matrixscience (http://www.matrixscience.com/) was used for protein identification by PMF. The following parameters were used for the database search: trypsin as the cleaving enzyme, a maximum of one missed cleavage, iodoacetamide (Cys) as a complete modification, oxidation (Met) as a partial modification, monoisotopic masses, and a mass tolerance of ±0.2 Da. PMF acceptance criteria are probability scoring. Detailed Mascot search parameters are summarized in Table S1.

### Cloning, expression, and purification of Os3BGlu6

#### Cloning and expression of Os3BGlu6

To isolate cDNA of rice GCase, total RNA was extracted from rice calli using the RNeasy Plant Mini Kit (Qiagen), and the mRNA was then purified using the Absolutely mRNA Purification Kit (Agilent) from the total RNA. Purified mRNA was subjected to cDNA synthesis using SuperScript III Reverse Transcriptase (Thermo Fisher).

The cDNA encoding the predicted mature Os3BGlu6 was amplified by PCR from synthesized cDNA with the specific primers, 5′-TGATTACGCCAAGCTGAAGGAGATATACATATGGCGCAGCAGAGCGGAGGA-3′ and 5′-CAGGCATG CAAGCTTCAGGTCTTCAGGAGGGC-3′.

Amplification was performed with 35 cycles of 94 °C for 2 min, 60 °C for 30 s, and 68 °C for 1 min, using KOD-Plus Neo (Toyobo). The DNA sequence of Os3BGlu6 gene isolated from *Oryza sativa*, L. cv. Nipponbare in this experiment was exactly the same with that was previously isolated from *Oryza sativa*, L. cv. Yukihikari (6). The PCR product encoding Os3BGlu6 was subcloned into pUC19 expression vector using In-Fusion HD cloning Kit (Takara) and the resultant plasmid was inserted into *E. coli* (DH5α). The strain was precultured for 16 h at 37 °C in 3 ml of Luria Bertani (LB) medium (1% tryptone, 0.5% yeast extract, and 0.5% NaCl, pH 7.0) containing ampicillin (50 μg/ml) and then cultured for 24 h at 37 °C in 150 ml of LB medium containing ampicillin. The cells were collected by centrifugation, and washed twice with buffer A. The washed cells were suspended in Buffer L and disrupted by sonication on ice. The sonicated solution was centrifuged at 36,000*g* for 60 min, and the supernatant was collected and filtered through a membrane filter (0.45 μm pore size). The filtrate was concentrated to 16 ml by Amicon ultrafiltration 30K (Merck Millipore) and then five times ultrafiltrated with Buffer I. Reaction mixtures with the recombinant Os3BGlu6 protein extracts expressed in *E. coli* and 100 μM glucosylceramide [d19:2(4*E*,8*E*,9Me)-C18h:1-GluCer] in 0.2 ml of Buffer J were incubated for 15 min at 37 °C. Before and after incubation, the reaction mixtures were analyzed by HPLC on ODS 120T column that was eluted with 81% ethanol at 50 °C (Fig. 1, *D* and *E*). A new peak liberated from the glucosylceramide was collected and confirmed as the ceramide [d19:2(4*E*,8*E*,9Me)-C18h:1-Cer] by LC-ESI-MS/MS (Precursor ion *m*/*z*: 592.6 [M+H]^+^, product ion m/z: 276.2).

#### Purification of recombinant Os3BGlu6

##### Step 1: purification of recombinant Os3BGlu6 using anion exchange chromatography

Part of the ultrafiltered sample (4 ml) from previous section was diluted to be finally 50 ml of 0.24% Triton X-100, 0.05% sodium cholate, and 20 mM sodium acetate (pH 6.0), then the 50 ml of diluted sample was subjected to FPLC (GE Healthcare) on a HiTrap Q HP column (5 ml × 3, GE Healthcare) equilibrated with Buffer C. After being washed with three-bed volumes of Buffer C, the column was eluted with a linear gradient from Buffer C to Buffer D at 3 ml/min. This protocol was done four times to purify 16 ml of the sample obtained in the previous section. The active fractions were pooled and concentrated to 4.5 ml using an Amicon ultrafiltration 30K (Merck Millipore), then ultrafiltered seven times with Buffer I.

##### Step 2: purification of recombinant Os3BGlu6 using cation exchange chromatography

Part of the sample (1.5 ml) obtained in Step 1 was diluted to be finally 30 ml of 0.2% Triton X-100, 0.05% sodium cholate, and 20 mM sodium acetate (pH 5.5); the 30 ml of diluted sample was then subjected to FPLC on a HiTrap SP HP column (5 ml × 2, GE Healthcare) equilibrated with Buffer E. After being washed with six-bed volumes of Buffer E, the column was eluted with a linear gradient from Buffer E to Buffer G at 3 ml/min. This protocol was done three times to purify 4.5 ml of the sample obtained in Step 1. The active fractions were pooled and concentrated to 4 ml using an Amicon ultrafiltration 30K (Merck Millipore), then ultrafiltered five times with Buffer I.

### Cloning and expression of soybean β-glucosidase 40

To isolate cDNA of soybean β-glucosidase 40, total RNA was extracted from soybean leaves using the RNeasy Plant Mini Kit. Total RNA was subjected to cDNA synthesis using PrimeScript II first strand cDNA Synthesis Kit (Takara). The cDNA encoding the predicted mature soybean β-glucosidase 40 was amplified by PCR form synthesized cDNA with the specific primers, 5′-TGATTACGCCAAGCTGAAGGAGATATACATATGATTCAGATATGCTCATCGG-3′ and 5′-CA GGCATGCAAGCTTTATTTAGTAGGTTTCAAGAAG-3′.

Amplification was performed with 40 cycles of 94 °C for 2 min, 60 °C for 30 s, and 68 °C for 1 min, using KOD-Plus Neo (Toyobo). The PCR product encoding soybean β-glucosidase 40 was subcloned into pUC19 expression vector using In-Fusion HD Cloning Kit, and the resultant plasmid was inserted into *E. coli* (DH5α). The strain was precultured for 16 h at 37 °C in 3 ml of LB medium containing ampicillin (50 μg/ml) and then cultured for 24 h at 37 °C in 150 ml of LB medium containing ampicillin. The cells were collected by centrifugation and washed twice with buffer A. The washed cells were suspended in Buffer L and disrupted by sonication on ice. The sonicated solution was centrifuged at 36,000*g* for 60 min, and the supernatant was collected and filtered through a membrane filter (0.45 μm pore size). The filtrate was concentrated to 16 ml by Amicon ultrafiltration 30K (Merck Millipore) and then five times ultrafiltrated with Buffer I. Reaction mixtures with the recombinant soybean β-glucosidase 40 protein extracts expressed in *E. coli* and 100 μM glucosylceramide [d19:2(4*E*,8*E*,9Me)-C18h:1-GluCer] in 0.2 ml of Buffer J were incubated for 30 min at 60 °C. Before and after incubation, the reaction mixtures were analyzed by HPLC on ODS 120T column that was eluted with 81% ethanol at 50 °C (Fig. 8). A new peak liberated from the glucosylceramide was collected and confirmed as the ceramide [d19:2(4*E*,8*E*,9Me)-C18h:1-Cer] by LC-ESI-MS/MS (Precursor ion *m*/*z*: 592.6 [M+H]^+^, product ion m/z: 276.2).

### Phylogenetic analysis of Os3BGlu6, the most similar protein in each plant and animal

The protein most similar to Os3BGlu6 was selected from each of plant and animal species using a BLASTp analysis (https://blast.ncbi.nlm.nih.gov/Blast.cgi). GenBank accession numbers of these proteins and GBA3 from human are as follows: XP_015628023.1, β-glucosidase 6 (Os3BGlu6, *Oryza sativa*, Japonica); VAI08420.1, unnamed protein (*Triticum turgidum* subsp. durum); PWZ58470.1, β-glucosidase 6 (*Z. mays*); XP_002465652.2, β-glucosidase 6 (*Sorghum bicolor*); RLN17293.1, β-glucosidase 6 (*Panicum miliaceum*); XP_004985215.1, β-glucosidase 6 (*Setaria italica*); KAE8788864.1, β-glucosidase 34 (*Hordeum vulgare*); XP_017616432.1, β-glucosidase 40 (*G. arboreum*); KAE8732915.1, β-glucosidase 34 (*Hibiscus syriacus*); XP_004228406.1, β-glucosidase 40 (*S. lycopersicum*); XP_016497770.1, β-glucosidase 40-like (*N. tabacum*); XP_006365136.1, β-glucosidase 40-like (*Solanum tuberosum*); XP_017224511.1, β-glucosidase 6-like (*D. carota* subsp. sativus); XP_011657400.1, β-glucosidase 40 (*C. sativus*); XP_008445465.1, β-glucosidase 40 (*C. melo*); XP_023754232.1, β-glucosidase 40 (*Lactuca sativa*); PRQ40673.1, β-glucosidase (*Rosa chinensis*); PQQ20207.1, β-glucosidase 40 (*Prunus yedoensis* var. nudiflora); VVA37410.1, β-glucosidase (*Prunus dulcis*); XP_003556662.1, β-glucosidase 40 (*G. max*); XP_019445904.1, β-glucosidase 40-like (*Lupinus angustifolius*); NP_173978.1, β-glucosidase 40 (*A. thaliana*); XP_013642074.1, β-glucosidase 40-like (*B. napus*); XP_013587431.1, β-glucosidase 40 (*B. oleracea* var. oleracea); XP_002967091.1, β-glucosidase 40 (*Selaginella moellendorffii*); PTQ42983.1, hypothetical protein (*Marchantia polymorpha*); XP_024362173.1, β-glucosidase 6-like (*Physcomitrella patens*); NP_001005742.1, GBA3 (*Homo sapiens*).

The sequences were aligned using multiple sequence comparison by log-expectation algorithm (44) implemented in MEGA X (45). The phylogenetic tree was constructed using MEGA X. Bootstrap values (more than 50%) at the nodes are based on 1000 replicates.

### Evaluation of defense response against disease in rice

The strength of the defense response induced by treatment with fungal glucosylceramide elicitor was assessed by measuring the amount of phytoalexins induced in rice plants using a modified method previously described (17, 18). Rice plants (*Oryza sativa* cv. Nipponbare) were grown in a greenhouse, and at the five-leaf stage, the surface of the fourth leaf was treated with 20 μl of 0.1% Tween 20 (control) or 0.1% Tween 20 plus 100 μM fungal glucosylceramide [d19:2(4*E*,8*E*,9Me)-C16h:0-GluCer] (elicitor). The amount of ceramide [d19:2(4*E*,8*E*,9Me)-C16h:0-Cer] produced and the total amount of phytoalexins (phytocassanes A–E plus momilactones A and B) (15, 16, 46) induced in the leaves 72 h after treatment with the control or elicitor solution were measured in the wild type and Os3BGlu6-deficient rice mutants (NE1537 and ND8040) using LC-ESI-MS/MS as described (26, 47).

### Statistics

For all data, comparisons were made using Student's *t* test. All data are shown as the mean ± SD with *p* < 0.05 considered statistically significant (∗*p* < 0.05, ∗∗*p* < 0.01).

## Data availability

All data presented in this article are contained within the article. The data for rice and soybean GCase genes have been deposited to DDBJ (DNA Data Bank of Japan). DDBJ accession numbers of rice and soybean GCase genes are LC515801 and LC515802, respectively. The MS proteomics data have been deposited to the Figshare repository (https://figshare.com/) with the dataset　identifier 10.6084/m9.figshare.16399194 for raw mass spectrometry data, 10.6084/m9.figshare.16400223 for the Mascot search results.

## Supporting information

This article contains supporting information (43).

## Conflict of interest

The authors declare that they have no conflicts of interest with the contents of this article.
