# Supplementary material for: Sphingadienine-1-phosphate levels are regulated by a novel glycoside hydrolase family 1 glucocerebrosidase widely distributed in seed plants
Source: J Biol Chem. 2021 Sep 23;297(5):101236. doi: 10.1016/j.jbc.2021.101236 (PMC8571087; doi:10.1016/j.jbc.2021.101236)
Supplement: Figure S1 and Tables S1–S3 [file mmc1.docx]

**Supporting information**

**Sphingadienine-1-phosphate levels are regulated by a novel glycoside hydrolase family 1 glucocerebrosidase widely distributed in seed plants**

Jinichiro Koga^1,＊^, Makoto Yazawa^1^, Koji Miyamoto^1^, Emi Yumoto^2^, Tomoyoshi Kubota^1^, Tomoko Sakazawa^1^, Syun Hashimoto^1^, Masaki Sato ^1^, and Hisakazu Yamane^1^

* For correspondence: Jinichiro Koga, jinichiro_koga@nasu.bio.teikyo-u.ac.jp

^1^Department of Biosciences, School of Science and Engineering, Teikyo University, Tochigi 320-8551, Japan

^2^Advanced Instrumental Analysis Center, Teikyo University, Tochigi 320-8551, Japan

**Figure S1.** PMF spectrum of 62-kDa protein

**Table S1.** Mascot search parameters.

**Table S2.** Protein sequence matches of 62-kDa protein for gi|254574853

**Table S3.** Information on peptides identified based on PMF spectral data of 62-kDa protein.


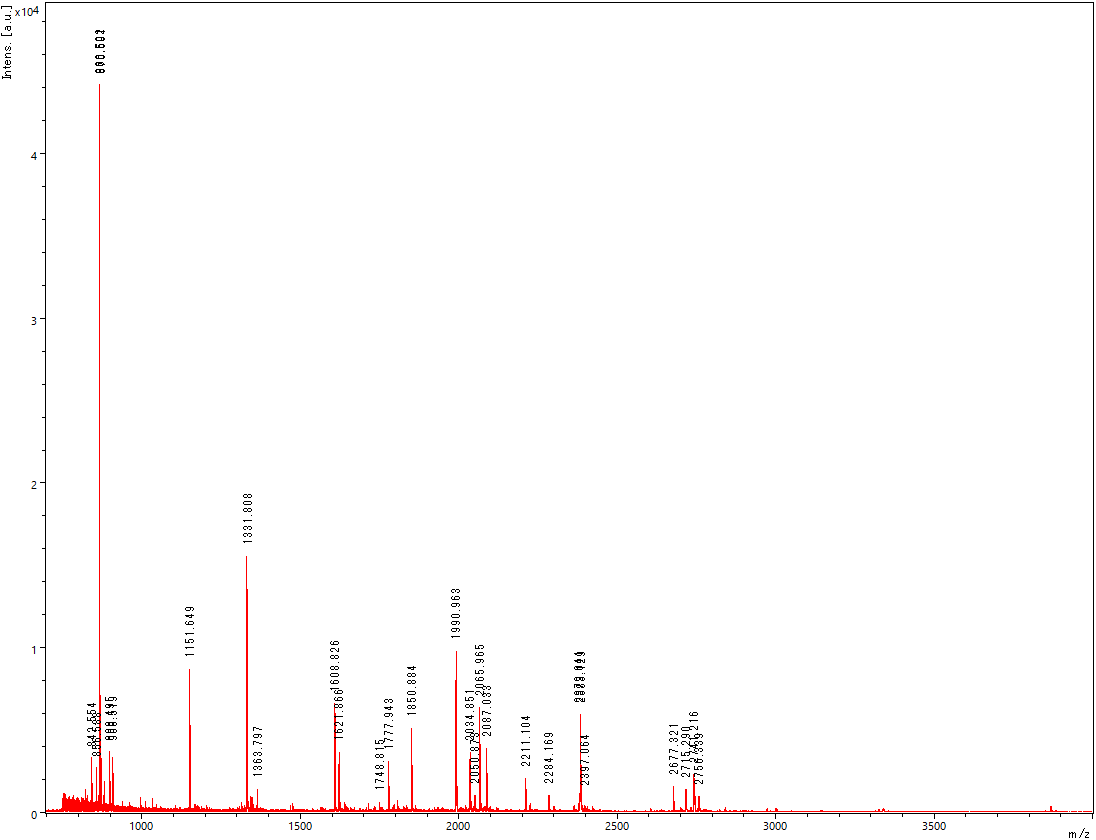
**Figure S1. PMF spectrum of 62-kDa protein.** For identification of components of the 62-kDa band on SDS-PAGE (Fig. 1F) by PMF with MALDI-TOF MS, the in-gel-digested protein sample was mixed with α-cyano-4-hydroxycinnamic acid in 50% acetonitrile and 0.1% TFA and subjected to MALDI-TOF MS.

**Table S1. Mascot search parameters**

Type of search PMF (Peptide Mass Fingerprint)

Search engine Mascot algorithm (2015/12/18)

Sequence database searched NCBInr

Release date of sequence database searched 2015/12/14

Taxonomy *Oryza sativa* (rice)

Number of entries in the database searched 159,631

Protease used to generate peptides Trypsin

Max missed cleavages 1

Fixed modifications Carbamidomethyl (C)

Variable modifications Oxidation (M)

Peptide mass tolerance ± 0.2 Da

Individual ions scores > 65

**Table S2. Protein sequence matches of 62-kDa protein for gi|254574853**

Number of matched masses 21

Number of unmatched masses 7

Protein sequence coverage 59%

Protein score 273 Expectation value 8e-23

**Table S3. Information on peptides identified based on PMF spectral data of 62-kDa protein**

Peptide sequence Precursor ion *m*/*z* Observed

Observed Mr (expt) Mr (calc) Delta modification

GSFPEGFVFGTASAAYQYEGAVK 2383.1292 2382.1219 2382.1168 0.0051 None

EDGRGQTIWDTFAHTFGK 2065.9653 2064.9580 2064.9654 -0.0074 None

GQTIWDTFAHTFGK 1608.8263 1607.8190 1607.7733 0.0458 None

ITDFSNADVAVDQYHR 1850.8836 1849.8763 1849.8595 0.0168 None

FEEDIQLMADMGMDAYR 2034.8515 2033.8442 2033.8533 -0.0091 None

FEEDIQLMADMGMDAYR 2050.8730 2049.8657 2049.8482 0.0175 Oxidation (M)

FSIAWSR 866.5023 865.4950 865.4446 0.0504 None

IYPNGVGQVNQAGIDHYNK 2087.0334 2086.0261 2086.0232 0.0029 None

LIDALLAK 856.5877 855.5804 855.5429 0.0375 None

GIQPYVTLYHWDLPQALEDKYK 2677.3209 2676.3136 2676.3588 -0.0452 None

QIVDDFAAYAETCFR 1748.8152 1747.8080 1747.7876 0.0204 None

AQEFQLGWFADPFFFGDYPATMR 2741.2164 2740.2091 2740.2421 -0.0330 None

GALDFVGINHYTTYYTR 1990.9634 1989.9561 1989.9585 -0.0024 None

ANSIWLYIVPR 1331.8076 1330.8003 1330.7397 0.0606 None

SLMNYVK 870.5914 869.5841 869.4317 0.1524 Oxidation (M)

YNSPPVYITENGMDDSNNPFISIK 2715.2905　2714.2832 2714.2534 0.0298 None

YHNDYLTNLAASIKEDGCDVR 2397.0640 2396.0568 2396.1066 -0.0499 None

GYFAWSLLDNWEWAAGYSSR 2379.0413 2378.0341 2378.0756 -0.0415 None

FGLYFVDYK 1151.6487 1150.6414 1150.5699 0.0715 None

FGLYFVDYKDNLK 1621.8660 1620.8587 1620.8188 0.0399 None

NSVQWFK 908.5186 907.5113 907.4552 0.0561 None

The search program Mascot (43) developed by Matrixscience (http://www.matrixscience.com/) was used for protein identification by PMF.
